# COVID-19 pandemic: an overview of epidemiology, parthenogenesis, diagnostics and potential vaccines and therapeutics

**DOI:** 10.4155/tde-2020-0035

**Published:** 2020-05-13

**Authors:** Haneen Amawi, Ghina'a I Abu Deiab, Alaa A A Aljabali, Kamal Dua, Murtaza M Tambuwala

**Affiliations:** ^1^Faculty of Pharmacy, Department of Pharmacy Practice, Yarmouk University, Irbid-Jordan; ^2^Faculty of Pharmacy, Department of Medicinal Chemistry & Pharmacognosy, Yarmouk University, Irbid-Jordan; ^3^Faculty of Pharmacy, Department of Pharmaceutics & Pharmaceutical Technology, Yarmouk University, Irbid-Jordan; ^4^Discipline of Pharmacy, Graduate School of Health, University of Technology Sydney, Ultimo, NSW 2007, Australia; ^5^School of Pharmacy & Pharmaceutical Sciences, Ulster University, Coleraine, County Londonderry, BT52 1SA, Northern Ireland, UK

**Keywords:** COVID-19, infection, repurposed therapies, SARS-CoV-2, vaccine, viruses

## Abstract

At the time of writing this review, severe acute respiratory coronavirus syndrome-2 (SARS-CoV-2) has infected more than 2,355,853 patients and resulted in more than 164,656 deaths worldwide (as of 20 April 2020). This review highlights the preventive measures, available clinical therapies and the potential of vaccine development against SARS-CoV-2 by taking into consideration the strong genetic similarities of the 2003 epidemic SARS-CoV. Recent studies are investigating the repurposing of US FDA-approved drugs as there is no available vaccine yet with many attempts under clinical evaluation. Several antivirals, antimalarials and immunomodulators that have shown activity against SARS-CoV and Middle East coronavirus respiratory syndromes are being evaluated. In particular, hydroxychloroquine, remdesivir, favipiravir, arbidol, tocilizumab and bevacizumab have shown promising results. The main aim of this review is to provide an overview of this pandemic and where we currently stand.

## Graphical abstract

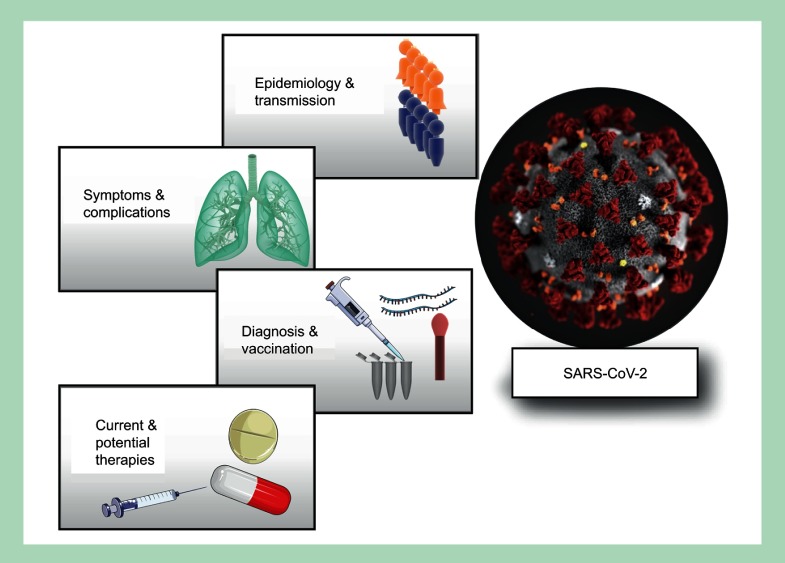

## Background

Viruses are defined as obligate intracellular parasites that infect cells susceptible and permissive to their life cycle [1]. Viruses have an amazing architectural arrangement with a protein capsid that protects the nucleic acid and forms from repetitive building units to give a mainly spherical arrangement of structures [2]. Viral diseases continue to appear and pose a major public health concern, according to the WHO. Many epidemics have been reported over the past 20 years, including severe acute respiratory coronavirus syndrome (SARS-CoV) from 2002 to 2003 and influenza A subtype H1N1 from 2010. Most recently, Saudi Arabia was initially infected by Middle East coronavirus respiratory syndrome (MERS-CoV), a member virus of the same family [3]. The latest coronavirus strain, severe acute respiratory coronavirus syndrome-2 (SAR-COV-2), is very infectious and highly contagious and spread across the globe very fast. WHO proclaimed the epidemic as an international disaster for the public safety of importance since it has expanded to over 18 nations. Four nations confirmed a human-to-human infection, at the meeting of 30 January 2020 under the International Health Regulations (IHR), 2005. The WHO announced this epidemic on 30 January 2020; a further milestone was established when the first case of the disease was recorded in the USA and not imported from China. This review highlights the prevention measures, available clinical therapies and the potential of vaccine development against SARS-CoV-2 [4,5]. Antivirals, antimalarials and immunomodulators that have shown to be active against SARS-CoV and MERS-CoV will be reviewed. Other potential therapies that have been suggested or proposed and are in the Research and Development process will also be discussed.

### Coronavirus family

Coronaviruses (CoVs) belong to the genus Coronavirus in *Coronaviridae* family. CoVs are enveloped, positive-stranded RNA viruses with a nucleocapsid (capsid with nucleic acid) reported size of 300–400 nm under the electron microscope [6]. All CoVs are pleomorphic viruses that usually produce 80–160 nm and 27–32 kb positive polarity of crown-shaped peplomers [7]. CoV recombinations are very large as RNA-dependent RNA polymerase (RdRP) jumps, and transcription errors are continually increasing, which might lead to genetic drifting within the same strain [8]. With their rapid mutation rates, CoVs are zoonotic viruses found in humans as well as other animal species, with a broad array of clinical symptoms from asymptomatic to the hospitalization in an intensive-care facility [3]. CoVs were not known to be highly pathogenic in humans until they were first detected in Guangdong in 2002 and 2003 with the severe acute respiratory syndrome (SARS) [9]. There were two more common types of CoVs, CoV-OC43, and CoV-229E, that trigger moderate infections in people with an adequate immune system, before these outbreaks. About 10 years ago, since SARS appeared, MERS-CoV in the Middle East countries, another extremely pathogenic CoV virus has evolved [9,10]. In December 2019, a novel coronavirus (nCoV) was established in Wuhan, Huanan, province of Hubei, and has become a significant global priority because of the outbreak of pneumonia, where livestock was exchanged (traded) [11]. The novel new virus SARS-CoV-2 is the seventh known CoV to infect humans from this viral family. At first, on 12 December 2019, an unexplained case of pneumonia was identified in Wuhan. Laboratory tests eliminated suspected influenza and other CoVs. On 7 January 2020, the authorities in China declared the isolation of the new CoV type [12]. On 12th January, 2019-nCoV was designated by WHO, and on 11 February 2020 was assigned COVID-19 name. A total of 2,355,853 recorded cases were registered, with 164,656 fatalities as of the 20 April 2020 [13]. On 29 January 2020, Li *et al.*‘s case study reported in the New Britain Journal of Medicine summarizes the 425 first documented cases in Wuhan [3,14].

Once the first case was identified, the infection was possibly spread from animal to human as a zoonotic agent. A second human-to-human route of transfer confirmed a surge in cases in Wuhan and globally after the shutdown of the Wuhan market and relocation of cases in China. At the time of writing this review, Although China declared that it is free of new cases, but have been reported at the beginning of April of 2020 of viral re-emergence. A surge in the reported cases in Europe (Italy, France, Spain and UK) and the USA had been reported [15]. This review is intended to provide an informed opinion on the epidemic and forms in which this initial outbreak may be handled and avoided with expert insight into the current and potential available therapies. Scientists at the Scripps Research Institute have found no proof that SARS-CoV-2 is a product of bio-engineering (manipulation) in laboratory facilities by conducting a comparative analysis of genomic data [16]. Therefore, this virus is possibly novel and subsequent natural results of the mutations that the CoVs family is renowned for having.

### Epidemiology of SARS-CoV-2

This epidemic has spread exponentially across the globe ever since the advent of the latest coronaviral epidemic COVID-19 triggered by the SARS-CoV-2 virus [17,18]. In consideration of the possibility of a pandemic, scientists and physicians have been trying to grasp this emerging virus and its pathophysiology to recognize potential therapeutic protocols and to find therapeutic agents and vaccinations that are successful in the disease management. Several pneumonia cases that were localized in Wuhan in December 2019 were identified, and sources were checked. On 12 December 2019, the first case of COVID-19 was identified with apparent pneumonia, while on 31 December 2019, 27 cases of extreme viral pneumonia were officially confirmed [15]. Etiological studies of people that came to the hospital due to specific viruses have been conducted. The medical history of these patients has increased the likelihood of a virus outbreak. Novel SARS-COV-2 from wild bats and group 2 β-CoVs, which comprises severe acute respiratory syndrome-related coronavirus (SARS-COV), was announced to be developed on 22 January 2020. This was the case, although COVID-19 and SARS belong to the same category of β-CoVs, genome-overlap between the two species is only 70%. The research group led by Bao S has reported that there are some genetic differences with SARS-CoV [17]. This outbreak has occurred in similar ways to the SARS epidemic during China's spring festival, which is China's most prominent traditional festival, where almost 3 billion citizens fly around the world to witness it. This created ideal conditions for the transmission and resulted in severe problems in the prevention and control of this extremely infectious disease. From 17 January till 23 February 2003, the Chinese Spring Festival ended in the SARS outbreak, and from 10th January to 18th February 2020, the festival was conducted once more. The number of COVID-19 reports from 10 to 22 January has risen rapidly. Wuhan is also a significant node of the spring festival transport network, the center of the outbreak, with approximately 10 million inhabitants. The number of tourists expected at the spring festival in 2020 rose 1, seven-times over 2003, the same festival, from 1.82 billion up to 3.11 billion. The massive population flow has often provided ideal conditions for this problem to spread [19].

About 2,355,853 cases were reported globally after the start of the outbreak, the evidence provided by the WHO safety specialist website (20 April at 22:51 CET), and 164,656 of these were deadly. Approximately 83,817 cases were recorded in China, where nearly all fatalities were registered in 4636. With the highest level of reported cases in USA at the time of preparing this review with around 759,687 reported cases and 40,682 fatalities on 20 April at 22:51 CET. The WHO current coronavirus board of directors (COVID-19) is the most modified source of outbreaks in this evolving pandemic that has updated statistics of the virus epidemiology.

### Virology pathogenesis

Based on the structural studies of α- and β-coronavirus, the viral genome encodes many structural proteins; including the spike (S) protein glycosylated, which serves as a critical inducer for the host immune response with a functional polybais (furin) cleavage site at the S1–S2 boundary through 12 nucleotides that have been reported [16]. The S protein mediates host cell invasion by SARS-CoV as well as SARS-CoV-2 through binding of the host cell's membrane protein receptor named angiotensin-converting enzyme 2 (ACE2) [6,20]. A detailed study conducted has shown that this cellular invasion includes the production of S-proteins, facilitated by serine protease TMPRSS211, formed by the host cell. The viral genome also encodes a variety of nonstructural proteins such as RNA-dependent RNA polymerase (RdRp), coronaviral principal protease (3CLpro) and papain-like protease (PLpro) [21]. The viral genome is discharged as a positive sense of a single-strand RNA (ssRNA) into the cell. Subsequently, it uses host cell protein translation machinery (ribosomes) to produce the viral polyprotein and then cleaves it into effector proteins through viral proteases 3CLpro and PLpro [22]. The most complex component of the CoV genome is the receptor-binding domain (RBD) in S protein. Six RBD amino acids were found to be important for ACE2 binding and the host spectrum of viruses close to SARS-CoV [23]. The Y442, S472, N479, D480, T487 and Y4911, which are L455, F486, Q493, S494, N501 and Y505 in SARS-CoV-2, are the co-ordinates centered on SARS-CoV. SARS-CoV-2 and the SARS-CoV are five or six residues. SARS-CoV-2 tends to have an RBD that connects humans, ferrets, cats and other highly-receptor-homologated animals with elevated ACE2 affinity [6,24].

### Sources & modes of transmission of SARS-CoV-2

As there were several new SARS-CoV-2 cases in association with the Huanan market in Wuhan [25,26], an animal host could be identified as a source of virus transmission. Since SARS-CoV-2 is identical to previous bat SARS-CoV [26], bats are possibly the host for their progenitor. While RaTG13, sampled with *Rhinolophus affinis* bat, is approximately 96% identical to SARS-CoV-2, indicating that it cannot effectively bind to human ACE2 [27]. Furthermore, illegally smuggled infected animals into Guangdong province, such as Malayan pangolins (*Manis javanica*), can carry CoVs that is identical to SARS-CoV-2 [28]. Besides, while there is no animal CoV quite close to SARS-CoV-2, the range of the CoVs is greatly undersampled in bats and other animals. In the S1–S2 junction of the CoV mutations, insertions and removals of nucleiotides may occur []. This demonstrates that a normal evolutionary cycle will lead to a polybasic cleavage location. In order for the precursor virus to obtain the polybase site of cleavage and the spike protein mutations necessary for the human binding of ACE2, it will possibly need an animal host with a large population density (for natural selection to occur effectively) and an ACE2 gene close to that present in human [16]. The SARS-CoV-2 progenitor has probably jumped to humans, gaining the mentioned genomic features by evolving during unrecognized human-to-human transmission. Such adaptations allowed the pandemic of the disease once it has been acquired [26,28].

SARS-CoV-2 infection is primarily thought to transmit from human-to-human among individuals nearby (about 6 feet) with each other through direct contact and droplets [17]. Such droplets may fall or be inhaled by a cough or sneezing onto the mouths or throats of surrounding people. Furthermore, it has been reported that people are known to be more infectious when they are most (sickest) symptomatic. Besides, an individual may get SARS-CoV-2 by contacting a surface or entity contaminated with the virus by rubbing ears, nose and maybe eyes after direct contact. The SARS-CoV-2 virus in some of the infected populations ‘community spread’ appears to be circulating quickly and sustainably among individuals [29].

### Symptoms of catching an infection

Information from public care bodies, reviews and guidelines provide for separating the clinical cases according to the seriousness of the clinical photos. The SARS-CoV-2 might be mild, moderate or severe based on the strength of the immune system of the infected individual. Acute influenza, ARDS, sepsis and septic shock are among severe health symptoms. A definite pattern in the bulk of cases tends to reflect the scientific development of the disease. In a proportion that has yet to be identified, after around a week, infected individual health outcomes have unexpectedly worsened, as respiratory failure has declined rapidly. The extreme respiratory failure conditions and medical requirements of sepsis and septic shock should be taken seriously [3].

Patients with uncomplicated (mild to moderate) illness usually have signs that include mild fever, dry cough, sore throat, respiratory irritation, fatigue, stomach aches and malaise, whereas reported dyspnea in patients were asymptomatic [15]. Nonrespiratory signs such as diarrhea are challenging to identify relative to prior HCoV infections. Moderate pneumonia respiratory signs such as cough and shortness of breath (or children's tachypnea, etc.) were reported for patients with some cases [13]. Extreme pneumonia fever is caused by heavy illnesses, respiratory depression or hypoxia (SpO2 <90% in the room). Fever is consistent with extreme pneumonia [30]. Nevertheless, fever signs thought to be correctly recognized as mild or sometimes missing, even in extreme cases of the disease. In children, cyanosis can occur. The description includes a psychiatric condition, and radiological terminology is used to remove complications with clinical and ventilatory requirements that are needed for this diagnosis [13]. This condition indicates a severe new respiratory problem and a deterioration of a respiratory feature that has already been established. The degree of hypoxia in various types of acute respiratory distress syndrome (ARDS) is distinct [31].

Furthermore, sepsis is a life-threatening organ dysfunction due to dysregulated host responses to suspected or proven organ dysfunctions [32]. The clinical pictures of patients with SARS-CoV-2 and sepsis are particularly severe, with a wide variety of signs, symptoms (cardiac disorders, such as extreme dyspnea and hypoxemia, abnormal vomiting, acidosis, altered mental state and functional organ changes), and signs of multi-organ shock presented as hyperbilirubinemia laboratory results [31].

### Clinical progression & diagnosis

The quest through Google dataset for SARS-CoV-2 diagnosis (as of 20 March 2020; summarized in Table 1) culminated in five policies, webpages with links and recommendations for common knowledge (WHO, Europe Center of Disease Control [CDC], US CDC, US FDA). Six diagnostic protocols using RT-PCR from six countries were released on the WHO website based on reverse transcriptase-polymerase chain reaction [31].

**Table 1.** SARS-CoV-2, SARS-CoV and MERS-CoV diagnose systematic assessment results.

Viral strain	Test	Samples	References	Ref.
SARS-CoV-2	rRT-PCR; E gene assay; confirmatory testing: RdRp gene assay	Respiratory samples from hospitalized patients		[33]
SARS-CoV-2	COVID-19 IgG/IgM rapid test kit	Saliva swab samples	Commercial kit from myLAB Box	
SARS-CoV-2	A colorimetric assay based on gold nanoparticles	Saliva swab	Oxford Suzhou Centre for Advanced Research (OSCAR)	
SARS-CoV-2	A colorimetric assay based on gold nanoparticles coated with glycans	Saliva swab	Iceni Diagnostics	
MERS-CoV	A collection of six separate, industrial, MERS CoV RNA detection kits focused on the PCR-RRT: (i) PowerChek (Kogene Biotech, Korea); (ii) DiaPlexQ (SolGent, Korea); (iii) Anyplex (Seegene), Korea) Screening: envelope gene (upE) Confirmation: ORF1a (iv) AccuPower (Bions, Korean) (v)	28 swabs alert for the other air viruses nasopharyngeal		[34]
MERS-CoV	Loopamp RNA Amplification Kit (RT-LAMP)	Laboratory isolates MERS-CoV swabs from healthy adults		[35]
MERS-CoV	A one-step rRT-PCR assay, based on specific TaqMan	Synthesis of UpE and ORF1b		[36]
SARS-CoV	Real-time qRT-PCR; ELISA technique	Samples obtained from 40-SARS hospitalized patients in Hong Kong		[37]
SARS-CoV	Enhanced RT-fluorescent PCR	Samples obtained from 80-SARS hospitalized patients in Hong Kong		[38]
SARS-CoV	Quantitative, RT and, nested PCR	Samples obtained from 46-SARS hospitalized patients from Taiwan		[39]
SARS-CoV	Western blot assay with N195 protein	274 clinical sera collected from patients with probable or suspected SARS, dengue fever, autoimmune diseases		[40]
SARS-CoV	RT-PCR	274 clinical sera collected from Hong Kong		[41]

#### Detection of genetic material (PCR)

Just as the SARS-CoV-2 appeared, real-time polymerase chain reactions (RT-PCR) remained the primary diagnostic tool for the latest viral strain among the different diagnostic platforms. One research addressed the usage of RT-PCR in diagnosing patients with SARS-CoV-2 from 16 experimental trials [33,42]. The respiratory tests were shown to be positive for the virus, although the serum in the early days of infection was negative. This has indicated that patients had elevated virus rates amid minimal signs in the early days of illness [43]. Besides commonly used RT-PCR for the diagnosis of MERS-CoV, several diagnostic procedures have been reported in four clinical trials, such as RT-LAM (RT-LAM)P, RT-insulated isothermal PCR (RT-IiPCR) and rRT-PCR (RT-PCR) as one-step test focused on unique TaqMan kit. RT-LAMP is as responsive as RT-PCR, as summarized in Table 1. It is also extremely sensitive and can be used for MERS-CoV strain identification. It is comparable to the standard diagnostic tests and is fast, easy and comfortable. RT-iiPCR and a one-step RRT-PCR study were equally responsive and demonstrated strong MER-CoV specificity. Finally, a review based on validating the six consumer RT-PCR kits was carried out [38,43].

The University of Hong Kong used two monoplex assays that were reactive Sarbecovirus (SARS-CoV-2, SARS-CoV and SARS coronavirus like) with coronaviruses [11]. Extracted viral RNA from SARS-CoV would be used as a supportive, positive control for the recommended procedure if SARS was successfully eradicated [37,44]. The N-gene is recommended to be used as the screening tool in RT-PCR analysis, whereas the Orf1b tool serves as the confirmative measure. The protocol has only been evaluated for the SARS-CoV RNA control samples. The synthetic oligonucleotide was used as a positive control. whereas, SARS-CoV-2 endogenous sequence is yet to be evaluated [44].

The US CDC distributed the real-time RT-PCR for the detection of SARS-CoV-2 with reaction primers and specifically engineered probes to detect SARS-like coronavirus in general. The same primer and protocol could be used for the detection of SARS-CoV-2 in particular based on high genomic similarities [15]. Additionally, aside from this protocol mentioned above, this protocol has not been evaluated on other platforms or reaction conditions chemistries. Each procedure has certain drawbacks. The analysis technique will be qualified and familiarized with and understood by the participating analysts. Inappropriate processing, distribution or treatment may result in false-negative results due to insufficient viral biological material in the sample. RNA viruses may also display major genetic variation and genetic drifts [45]. This may lead to a discrepancy between the PCR primers and the detection probe sequence that may decrease the test efficiency or contribute to false-negative outcomes [45]. The point of care (POC) evaluation kit can presumably mitigate such limits, that should be given the highest priority in the next few months for research and development of diagnostic kits to increase the sensitivity and the reliability of the test [15]. It may contribute to a discrepancy between the first and the goal series sensors, which may reduce the test output or contribute to incorrect adverse outcomes. The point of care test kit can mitigate these limitations, which should be highly prioritized in the next few months for research and development [15].

#### Serological testing

CDC is said to have a two-serological study procedure, by way of two screening tests and a confirmation check to identify MERS-COV antibody [15]. Enzyme-linked immunosorbent assay (ELISA) is a screening procedure used to determine the existence and amount of particular antibodies (nucleocapsid (N) and spike (S) ) that are bound to a viral protein [33,45]. If either ELISA confirms whether a clinical sample is antibody-positive, the CDC recommends the microneutralization method to validate the positive tests [7,15]. The microneutralization procedure is an exact serological method designed to evaluate neutralizing antibodies or antibodies which may neutralize the virus. This procedure is considered a gold-standard for the identification of SARS-CoV-2 antibodies in serum samples. The microneutralization method, though, relative to ELISA, is time-consuming and labor-intensive, require at least 5 days to produce results [15]. FDA has approved the first fast test by cepheid for the latest coronavirus on 23 March 2020 [46].

The level of contamination, including measurement of asymptomatic incidence and the attack intensity, is easily calculated through serological measures including ELISA, IIFT, and neutralization studies. The serological experiments identify proteins and antigens in addition to the identification of the virus genome using biochemical approaches [45]. There is a pause since the virus usually aimed at anti-corps from 14 and 28 days after the start of the disease. Furthermore, evidence indicates that low titers of antibodies may be correlated with high viral load in a second week or with delayed antibody development. Serological diagnosis is more likely to be employed if nucleic amplification tests (NAAT) are not given or available [30].

### Structural proteins as vaccine candidates

**Figure 1.**
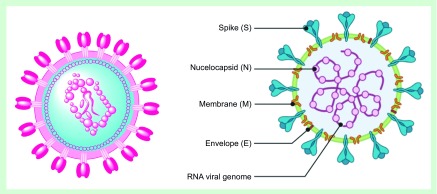
Schematic drawings of the SARS-CoV-2 structure showing the exterior envelop with its distinctive spike glycoprotein S, the virus capsid that is protecting the nucleic acid inside and the M and E proteins. The right image was generated on ChemDraw.

The first and complete genome sequence of SARS-COV-2, which provides the key to the possible structure and glycosylation pattern of viral proteins and thus modes of association with a host cell, has been recently reported (25 January 2020) NCBI (GenBank: MN908947.3) [47]. This is an essential step in developing a vaccine for SARS-COV-2. All the coronaviruses, including SARS-CoV-2, encodes nucleocapsid protein (N protein), and S protein [20], as shown in Figure 1. The N-Protein is a structural protein bound to the RNA genome of coronavirus and thereby forms a capsid around the enclosed nucleic acid. The N-protein has the following roles in viral life-cycle: associates with the viral membrane protein during viral assembly, and facilitates the production and folding of RNA, this plays a part in viral budding. S protein has two main tasks that enable host infection: it facilitates the interaction between viruses and host cells throughout the surface receptors and it enables their entrance into the host's cell by helping to connect the viral cell membranes with the host's membranes [20,47]. ACE2 is an intrinsic protein membrane that enables the entry of SARS-COV-2 throughout invasion by binding its extracellular peptidase domain to S protein [26].

The glycoprotein of the outer membrane, renowned for its glycosylation, is identical to many other coronaviruses [20]. The primary host interacting protein is essential for cell adhesion (such as ACE2, CD26, Ezrin, cyclophilins and other cell adhesive factors). The specific host cell factors or proteins that make the current SARS-CoV-2 simpler are, therefore, still elusive [48]. Therefore the undertaken research at the present moment to investigate SARS-CoV-2 spike structures for glycoprotein and glycan shield patterns that have significant effects on viral camouflage and mode of cell entry, which could help the production of new infection screening, vaccinations and treatment. Another report of the COVID-19 and SARS-CoV clustal/w sequential coordination of glycoproteins reveals a 91% similarity in the S2 domain region (aa570–aa1278), but in three other regions (aaa677–690, wing) (aaa877–884 and aa930–943, stalk), there are no structural correlations. However, in the S1 domain, with a 51% difference in (aa01–aa550), known for its host–cell interaction involving cell adhesion and virulence [49]. The potential molecular associations between S protein of SARS-CoV-2 and human CD26 receptors are considered to investigate structural variations or similarities in the interaction between SARS-CoV and SARS-CoV-2 S protein.

**Figure 2.**
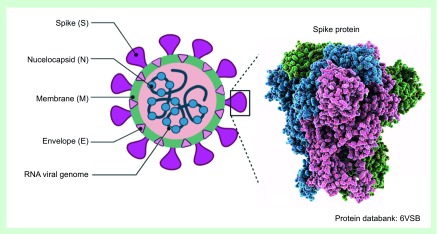
Schematic representation of the novel SARS-CoV-2 viral capsid. The coronavirus spike (S) protein mediates membrane fusion by binding to cellular receptors. With a magnification of S protein from the PDP with the ID entry 6VSB. The schematic drawings were generated from Biorender online application.

To this end, the researchers used Cluspro-protein-docking (www.cluspro.bu.edu) and Frodock (http:/frodock.chaconlab.org) servers for establishing a model of SARS-CoV-2 spike and human CD26 model to predict the amino acids involved in the binding between the S1 domain and the CD26 receptor as shown in Figure 2 [49].

The N-terminal S1 domain of spike glycoprotein, interacting with several host cell proteins, is the primary motive for the trafficking in and the hijacking of coronaviruses in the host cells [20]. Host cellular receptors (CD-26) is the crucial component in the immune regulatory pathway of viral infections responsible for the cleavage of amino-terminal dipeptides from polypeptides with either L-proline or L-alanine in the penultimate position of the sequence [13]. As SARS-CoV-2 has gained global interest, some 15–20 possible vaccine candidates worldwide have been in the pipeline utilizing a range of technologies (e.g., messenger RNA, DNA, nanoparticles, synthetic and modified virus-like particles). It can take more than 1 year to complete clinical trials for most candidates with the exception of those sponsored by the Coalition for Epidemic Preparedness Technologies (CEPI). However, the BGI kit met the national medicinal devices' authorization test and is actually in use in China's therapeutic and testing centers [50]. Out of 570 original research reviewed for SARS-CoV-2, SARS CoV or MERS-CoV vaccinations, only four have been included [3]. In the majority of the SARS and MERS research, as carried out in cell or animal models, they were omitted. The four trials used in this analysis included the Phase-I SARS or MERS clinical tests. No population style studies were carried out at the screening point on the SARS-CoV-2 (cell, species, human). The clinical trials released were conducted mostly in the USA, but one on SARS in China [51–53]. Both SARS and MERS vaccine candidates have been documented to be healthy, well-tolerated and capable of stimulating appropriate and suitable immune response among participants.

Moreover, another research paper illustrates six clinical trials of Phase I listed in the list of Clinicaltrials.gov [54]. The health and immunogenicity of their respective MERS-CoV vaccine applicants are both checked but have yet to be released [55]. The trials will be finished by December 2020 (two Russian studies) [54] and December 2021 (German study) [54]. Medicago company has announced the development of a plant-based vaccine for the SARS-CoV-2 release in March 2020. Besides, further attempts for vaccine development from Moderna utilizing mRNA platform, Inovios DNA vaccine and College of Medicine and University of Queensland's utilizing subunit recombinant expression of potential vaccines [56–63].

### Essential proteins & their roles in viral infection

SARS-CoV-2, based on full-length phylogenetic genome review, is found to be similar to SARS-CoV much more than MERS-CoV [16]. At the level of each structural protein (S, E, M and N), the findings are reported here. Indeed this was supported by a direct comparison sequence-based study that SARS-CoV-2 and SARS-CoV proteins are genetically identical in their M, N and E protein, although S protein has dramatically reduced its reproductive similarity (but still high). In this same study, the resemblance of SARS-CoV-2 and MERS-CoV for both proteins were reported, on the other hand, was considerably lower, which is also apparent from the phylogenetic trees involved [20,28 31].

The spike glycoproteins composed of two subunits (S1 and S2) are among the structural components of CoVs. The spikes on the viral surface are produced by S protein homo-trimers, which direct the connection to the host receptors, as depicted in Figure 2. The S2 domain, including a fusion peptide, a transmembrane domain and a cytoplasmic domain – is firmly retained in SARS-CoV-2 [27]. It may also be a candidate for antiviral agents (anti-S2). On the other side, only 40% of the SARS-CoV-2 produces an amino acid identity in the spike receptor-binding domain [3,28]. Researchers published a variety of SARS-CoV-2 gene sequences in foreign gene banks such as GenBank. This gene mapping is essential to allow researchers to track the phylogenetic tree of the virus and, particularly, to identify strains that differ by mutation. A spike mutation possibly occurred at the end of November 2019 caused jumping to human beings, according to recent studies. Ciccozzi *et al.*, in particular, contrasted the gene sequence of the SARS-CoV-2 to the genome of Sars-CoV [3,64].

### Prevention

The latest approach to minimize the distribution of incidents is proactive steps. Thanks to a growing outbreak when R0 approaches 1 (SARS-CoV-2 is 2.2), regulation strategies need to work on reducing the value down to less than 1 [13]. Preventive approaches rely on hospital separation and diligent infection management, including effective preventive intervention and emergency treatment for an affected hospital. For starters, during the processing of specimens, droplets, touch and airborne measures should be taken, and the usage of sputum induction prevented. The WHO and other organizations have made the following general guidelines: prevent intimate contact with people having severe respiratory infections. Always wash hands, especially after being in contact with contaminated individuals or environment. Stop unsafe field touch or wildlife interaction. People with severe airway diseases should remain away, coughs or sneezes should be protected with jetty tissue or fabric and their hands should also be cleaned. Strengthen the implementation of strict hygiene measures to avoid and manage infections, particularly in the emergency medicine departments [17]. Immunocompromised individuals should not attend public meetings. The most effective technique is to use handheld sanitizer, wash hands, avoid interaction with face and mouth after engaging in contaminated areas. Infected caregivers should use PPE, gloves, eye cover, gowns and face mask (N95 or FFP3) to avoid the spread of the pathogen.

### Emerging diseases in the age of social media

News of SARS-CoV-2 spread over the internet with broad coverage, and social media transported the news of the disease much faster than previously reported. Over the years, websites such as FluTrackers.com, ProMED (promedmail.org), and others have allowed disease knowledge to be gathered from all around the world and encouraged its distribution to stakeholders. MERS-CoV first attracted the attention of scientific researchers, virologists and public health authorities as to the novel coronavirus embedded in ProMED Mail in 2012 and afterward [11]. 8 years later, a closer related network rapidly circulated claims regarding potential causes from the Wuhan municipal health board. Early in an epidemic, rumors with elements of fact and baseless paranoia can hardly differentiate. The linguistic barriers and record sources may intensify this reality. In this case, however, speculation of a new coronavirus was fueled by well-formulated statements that explicitly excluded individual families of viruses (influenza, adenovirus) but exclusively excluded coronavirus SARS-CoV and MERS-CoV. Following SARS’ experiences, others became worried that the facts should be preserved. The planet behaved both with fear and alleviated as the agent was ultimately identified as a SARS-CoV-2, the epidemic will not be cached. Although far from flawless, the government's reaction to SARS-CoV-2 compares strongly with the early SARS epidemic. The rapid release of SARS-CoV-2 sequences provided for rapid interaction, review and the creation of diagnostic tests within the scientific community [16]. Both the Chinese CDC and Wuhan municipal health board have frequently reported reports of the incidents and patient condition to the public health officials so that they can track the situation in real-time. Online media also enabled researchers from all over the world to link up-to-date sequential details and illustrate critical disease acknowledgements. The opportunity to exchange news reports and pieces of evidence in real-time with experts and public health professionals around the globe does not necessarily deliver accurate findings, and it is a massive improvement in the approach to outbreaks. Such openness has rendered the global science community aware of new partnerships and reacted rapidly. While several unknowns remain present with SARS-CoV-2, the environment is dedicated to fighting the SARS-CoV-2 virus imminently. Perhaps this indicates that the experiences from the epidemic of SARS were learned.

## Current treatments adapted to manage the disease

Until the time of this review, there are no specific therapeutic regimens approved for treating SARS-CoV-2 infection. The development of novel compounds or vaccines that work correctly against SARS-CoV-2 is a time-consuming process. Thus, efforts are focusing on repurposing the use of drugs available on the market to act against SARS-CoV-2 [65–67]. Chloroquine and hydroxychloroquine stand as excellent examples and are being adopted at the moment in the standard clinical practices of China for SARS-CoV-2 infection [27]. However, the validity of using these compounds should be further confirmed. Accordingly, agents with confirmed curing potentials are still lacking. All the therapeutic agents are still under evaluation, and the outcomes derived from clinical trials will determine future winners of the race. Currently, patients are still managed adjunctively. Standard care is composed of isolation and prevention measurements, supportive care for symptoms and complications as well as advanced organs supports in patients with severe illness status [68]. The patients with mild illness and no risk factors can be managed in outpatient settings. However, due to risks of deterioration in health, sudden respiratory failure and isolation failure, inpatients setting are preferred when possible. Outpatients settings include sporadic cases or small clusters, or in repurposed, nontraditional settings; or at home [69]. The isolation and prevention measurements include isolating patients and all suspected cases in a separate area. The isolation should be continued for at least 2 weeks after symptoms relief [70].

The supportive care includes oxygen therapy, conservative fluids supply, managing complications according to what each patient develops, empirical antimicrobial drugs, antipyretic/analgesics, mechanical ventilation and corticosteroids if indicated for other reasons [70]. Oxygen therapy is indicated at a rate of 5 L/min to counteract respiratory distress, hypoxemia or shock. It should be continued to reach target oxygen saturation >94% during resuscitation, >90% in stable cases for most patients and >95% for pregnant women. Mechanical ventilation should be given for patients with severe deterioration in respiratory functions such as acute respiratory distress syndrome (ARDS) [43]. Complications should be expected, including sepsis, acute respiratory distress syndrome, septic shock, acute kidney injury, acute cardiac injury, acute liver injury and should be managed according to their protocols. Empirical antibiotics should be given based on local epidemiology, common pathogens and discontinued after lab tests.

Antipyretic/analgesics should be prescribed as needed for pain and fever and should not be administrated on routine bases. These agents might mask fever and delay diagnosis and treatment [71]. Both paracetamol and NSAIDs can be considered to relieve pain. The reports for avoiding ibuprofen are not validated yet [69,70]. These reports suggest that NSAIDs may upregulate ACE receptors, which could worsen the disease progression [71]. Examples of supportive management are described briefly in several studies. For example, 199 patients in a clinical study conducted in Jin Yin-Tan Hospital, received a supportive therapy of supplemental oxygen, noninvasive and invasive ventilation, antibiotics, vasopressor support, renal-replacement therapy and extracorporeal membrane oxygenation (ECMO) [72]. Another clinical study at the national clinical research center for infectious diseases (the Third People's Hospital of Shenzhen), Shenzhen, China, has reported the use of oxygen inhalation, oral or intravenous rehydration therapy, electrolyte correction, antipyretics, analgesics and antiemetic drugs as supportive therapy for the patient [73]. Another example of supportive care is what is given to the first diagnosed case of SARS-CoV-2 in Washington, USA. The dosage of 650 mg acetaminophen every 4 h and 600 mg ibuprofen every 6 h was administered as prescribed to the patient. In the first 6 days of hospitalization, he was also given 600 mg guaifenesin for persistent coughing and around 6 L of regular saline [74]. As mentioned above, the used empirical antibiotics are varied depending on local epidemiology and common bacterial pathogens [17]. For example, a retrospective study from Jinyintan Hospital in Wuhan, China showed that antibiotics were given to 70 patients (71%). About 25 patients received a single antibiotic, while 45 patients received a combination of antibiotics. Used antibiotics included cephalosporins, quinolones, carbapenems, tigecycline and/or linezolid [75]. Another study on 139 patients has shown that antibacterial therapy is given as moxifloxacin, 64.4%; ceftriaxone, 24.6%; and azithromycin, 18.1% [76]. There are still no detailed guidelines for SARS-CoV-2 patients with comorbidities, including cardiovascular diseases, asthma, and cancer. However, current recommendations are still according to the conventional guidelines for each comorbidity [70].

## Repurposing drugs for SARS-CoV-2 (drugs in clinical trials)

As discussed above, the repurposing of existing drugs is the fast solution to act against the invasive spread of SARS-CoV-2 infection. Several drugs have been used before to control and treat previous viral outbreaks, including the SARS-CoV outbreak in 2003 and the MERS-CoV outbreak in 2012, which are currently being investigated to determine their efficiency in improving patients' survival and reducing the viral load of SARS-CoV-2 infection [77]. The investigations include antiviral drugs such as lopinavir, ritonavir, favipiravir, ribavirin and remdesivir [78]. Investigated drugs also include antimalarials, immunomodulators, VEGF inhibitors, corticosteroids, among others. Several of these medications are now part of the recommendations of the People's República of China National Health Commission (NHC) for the study of SARS-CoV-2-induced pneumonia for prevention, diagnostic and care [78]. *In silico*, *in vitro* and clinical studies are intensively conducted throughout the world, especially in China and USA. For example, molecular modeling studies are using docking software to determine the binding efficiency of these compounds to SARS-CoV-2. These studies are aiming to validate the repurposing of the use of different drugs such HIV protease inhibitors, nucleoside analogs for SARS-CoV-2 and other existing drugs with antiviral activity [79].

### Antiviral agents

Lopinavir (LPV) is a HIV type 1 aspartate protease inhibitor while ritonavir (RTV) is usually combined to it to increase the plasma half-life of LPV by inhibiting CYP450 enzyme [14]. Since the outbreak, several clinical trials have been investigated on the potentials of this combination (LPV/RTV) on SARS-CoV-2 patients outcomes. A clinical trial was conducted in Jin Yin-Tan Hospital, Wuhan, on 199 seriously ill patients of SARS-CoV-2 infection [80]. Male and nonpregnant patients of 18 years or older were included. The patients have an oxygen saturation of 94% or less with pneumonia confirmed by chest imagining. They were divided into two groups: a control group received the standard care in hospital, and the other treatment group received a combination of LPV/RTV (400 and 100 mg, respectively) twice daily plus the standard hospital care for 14 days. The treatment group showed no improvement in survival compared with control patients. The mortality percentage in LPV/RTV patients was not significantly different from control 19.2, and 25%, respectively [81]. No differences in the percentages of viral RNA detection was found at different times points in the members of the two groups [72]. Another clinical trial was conducted at the Third People's Hospital of Shenzhen to measure the effectiveness of favipiravir (FPV) compared with LPV/RTV combination as control. FPV is a novel RNA-dependent RNA-polymerase (RdRp) inhibitor that showed promising *in vitro* results on SARS-CoV-2 [82]. It blocks the replication of several viruses other than influenza. The included patients have an age range of 16–75. Patients with severe conditions, including RR >30, oxygen saturation <93%, respiratory failure, shock, and end-stage kidney or liver diseases, were excluded. The FPV group included 35 patients and received FPV day 1: 1600 mg twice daily; days 2–14: 600 mg twice daily) plus interferon alpha (IFN-α) by aerosol inhalation (5 million U twice daily). The LPV/RTV group received (days 1–14: 400 mg/100 mg twice daily) plus IFN-α by aerosol inhalation (5 million U twice daily). Standard care was given to both groups. Clinical outcomes include viral clearance (two constitutive negative results on qPCR detection throughout 24 h), changes in chest imaging (improvement in CT scan for lung parenchyma based on well-defined scales), as well as adverse drug effects (by questionaries and lab results). The median time of viral clearance was significantly lower in FPV group compared with the LPV/RTV group 4 and 11 days, respectively. The improvement rate in CT scans was only higher in FPV group on day 14 of the treatment compared with LPV/RTV group 91.4 and 62.2%. The FPV group showed fewer adverse drug reactions compared with LPV/RTV group, and no patients needed to discontinue the treatment [73]. Therefore, FPV stands as a promising agent in the management of SARS-CoV-2. Currently, three clinical trials are being conducted to validate further the role of FPV in the management of SARS-CoV-2 infection (NCT04303299, NCT04310228 and NCT04273763). FPV is being investigated as monotherapy or in combination therapy. It is also being compared with placebo or other antiviral regimens. Examples of combinations are LPV/RTV plus FPV, darunavir/ritonavir, chloroquine and FPV combined with tocilizumab [83].

Clinical trials are also being conducted on the antiviral drug, remdesivir (RDV) (Phase III), after reports on its significant effect when given intravenously to some SARS-CoV-2 patients [77]. RDV is a broad-spectrum antiviral agent by acting as a nucleoside analog that was initially developed to treat Ebola. A molecular modeling study suggested that RDV could be a potential therapeutic agent as the active form (CHEMBL2016761) of RDV has shown perfect docking scores among other antiviral agents [79]. It also showed promising *in vitro* activities by blocking the viral infection of SARS-CoV-2, as demonstrated by Wang M. *et al.*, (EC_50_ = 0.77 μM; CC_50_ >100 μM; SI >129.87) [84]. A clinical case study for the first patient diagnosed with SARS-CoV-2 infection in USA has shown promising effectiveness of RDV [74]. Infusion with RDV was administrated to patients on day 7 after developing worsening in clinical conditions (pneumonia). The patient showed improvement in clinical symptoms, oxygen saturation and CT imaging scans [74]. Thus, RDV is currently one of the most promising antiviral agents for reversing SARS-CoV-2 infection. Currently, about seven clinical trials are registered for investigating RDV at Clinicaltrials.gov and being conducted in USA and China (NCT04292899, NCT04292730, NCT04252664, NCT04257656, NCT04280705, NCT04315948 and NCT04302766). The studies have differences in patients disease conditions (mild, moderate and severe), treatment durations (5, 9 and 10 days), as well as the control group (RDV is being compared with placebo, LPV/RTV or LPV/RTV plus IFN-ß-1a) [85]. The safety profile for RDV is acceptable with GI symptoms and has the most common side effects, including nausea, vomiting and rectal bleeding. Some patients also experience elevations in liver enzymes [86]. A very recent case study was published showing the advantage of delayed administration of RDV to patients with severe SAR-COV-2-associated pneumonia. The patient received RDV on day 13 of symptoms and showed significant improvement in respiratory symptoms (extubation) after 6 h of RDV first dose [87]. An NIH-supported clinical trial is being conducted in the USA. RDV will be provided to patients as a loading dose of 200 mg on the first day of the study. Then, the drug will be given as 100 mg in the following days for 10 days. The patients' clinical outcomes from full recovery to death will be compared with the placebo group [88].

Ribavirin (RBV) is another broad-spectrum antiviral agent and acts as a nucleoside analog [78]. *In vitro* cytotoxicity of RBV against SARS-CoV-2 was evaluated with EC_50_ = 109.5 μM [89]. RBV is being given for SARS-CoV-2 patients in different hospitals. However, the clinical outcomes from its administration are still not clear. RBV was given to 80 patients referred to the First People's Hospital of Yancheng City, the Second People's Hospital of Yancheng Cit and the Fifth People's Hospital of Wuxi from 22 January to 14 February 2020, in China. RBV was given intravenously for periods of 3–12 days [28]. It is recommended to be given at a dose of 500 mg each time, two- to three-times/day, in combination with other drugs such as IFN-α or LPV/RTV [90]. Currently, one clinical trial (NCT04276688) is recruiting patients to determine the efficacy of RBV for SARS-CoV-2 infection at the University of Hong Kong, Queen Mary Hospital. In the study, RBV (400 mg twice daily for 14 days) was administrated in addition to LPV/RTV and INF beta-IB compared with the control group with LPV/RTV alone [91].

Arbidol is another antiviral that is being considered for SARS-CoV-2 infection. A retrospective study from the Fifth Affiliated Hospital of Sun Yat-Sen University has shown that the addition of arbidol to or LPV/RTV treatment significantly provided additional benefits on viral clearance and patients' clinical outcomes. Arbidol was administered at 200 mg every 8 h for the entire study group of 16 patients and LPV (400 mg)/RTV (100 mg) orally every 12 h until coronavirus had been reported three-times with negative results, while the control group (17 patients) received and LPV (400 mg)/RTV (100 mg) orally every 12 h. The combination group achieved higher viral clearance rates at days 7 and 14 compared with the control group (75 and 94% vs 35 and 52.9%, respectively). LPV/RTV, combined with arbidol, have shown antiviral effects in SARS-CoV-2 in other reports from the First Affiliated Hospital of Zhejiang University School of Medicine [92]. This is maybe important as earlier viral clearance is associated with more prevention of severe lung lesions. Indeed, the patients' CT imaging at day 7 was significantly better in a combination group compared with control (69 vs 29%, respectively) [93]. Current ongoing clinical trials are further investigating the role of arbidol in the management of SARS-CoV-2 pneumonia in different combinations, including NCT04260594 and NCT04286503 [94]. Clevudine is another antiviral agent that is currently being investigated in Phase II clinical trial for its potentials against SAR-CoV-2 [95].

### Antimalarial agents

Chloroquine (CQ) is currently being investigated extensively for its promising activity against SARS-CoV-2 infection [96,97]. CQ is an old antimalarial drug with limited use due to its resistance as well as poisoning risk (rhythm disturbance, QT interval prolongation) [98]. The derivative hydroxychloroquine (HCQ) was developed later and showed better clinical safety and lower risks of toxicities [99]. HCQ is also an option for the management of autoimmune diseases, including rheumatoid arthritis (RA) and systemic lupus erythematosus. HCQ is a highly available drug with a low cost and an acceptable toxicity profile [80]. Moreover, HCQ has good oral bioavailability allowing it to reach significant blood concentration that is sufficient to inhibit SARS-CoV-2. These properties nominate HCQ as a great candidate to be applied on large scale use such as the SARS-CoV-2 outbreak. On the *in vitro* level, HCQ showed significant inhibition of the SAR-CoV-2 infection [46]. CQ and HCQ resulted in significant antiviral cytotoxicity in African green monkey kidney VeroE6 cells (ATCC-1586) (CC_50_ 273.20 and 249.50 μM, respectively). The two compounds reduced the viral RNA copy number, with CQ being significantly more potent [100]. Another *in vitro* study has shown the potency of CQ and HCQ against SARS-CoV-2 with HCQ with enhanced potency (EC_50_ = 0.72% μM vs 5.47% μM) [101]. Suggested mechanisms of action include interfering with the pH-dependent steps of viral replication by increasing the pH of intracellular vesicles such as lysosomes and endosomes [96]. CQ also blocks the ACE2 receptor glycosylation and thus prevents the S protein binding [102]. Wang *et al.* have demonstrated potent blocking of SARS-COV-2 viral infection by CQ (EC_50_ = 1.13 μM; CC_50_ >100 μM, SI >88.50) [84]. CQ and HCQ also have immunomodulatory effects that might help in reversing the hyperinflammation and cytokine storm associated with SARS-COV-2 pneumonia [102].

On the clinical levels, until 22 March, several active clinical trials are currently conducted in China according to the Chinese Clinical Trial Registry to test the CQ or HCQ efficiency on SARS-CoV-2 infection [97]. The clinical outcomes include time to clinical recovery, all-cause mortality, length of hospital stay, length of ICU stay, relapse after discharge, liver function tests, C-reactive protein (CRP), the incidence of adverse effects, days on mechanical ventilation, cost, among others. These trials are investigating CQ or HCQ alone or in combination with other therapies. Some of these trials published its outcomes, and some are still waiting for the results. Reports from over 100 patients in studies demonstrated that CQ showed excellent results compared with control. CQ treated patients showed lower viral load, better CT imaging, and shorter disease period [97]. The ongoing clinical studies, efficacy and safety of CQ or HCQ alone in treating SARS-CoV-2 are being investigated in ChiCTR2000030054 (a total 80 patients are enrolled), ChiCTR2000029992 (three groups: CQ, HCQ and control), ChiCTR2000029988 (severe patients/critical illness cases), ChiCTR2000029975 (CQ is given as aerosol inhalation), ChiCTR2000029935 (100 patients), ChiCTR2000029899 (HCQ vs CQ), ChiCTR2000029898 (HCQ vs CQ), ChiCTR2000029803 (HCQ as prophylactics to prevent SARS-CoV-2), ChiCTR2000029741 (CQ vs LPV/RTV), ChiCTR2000029559 (HCQ vs placebo) and ChiCTR2000029542 (CQ vs placebo) [103].

In parallel, studies are being conducted all over the world, investigating CQ and HCQ effectiveness in the prevention and treatment of SAR-CoV-2. Clinical trials were registered to the Clinicaltrials.gov from USA, Korea, Mexico, among others [104]. Interestingly, a clinical prevention study (COPCOV) is intended to administrate CQ in healthy, not previously infected by SAR-CoV-2 volunteers. CQ is to be initially given as a loading dose of 10 mg base/kg, followed by 150 mg daily (250 mg CQ phosphate salt) will be taken for 3 months [104].

Combinations of other drugs with CQ are also being investigated in ongoing clinical trials. FBV tablets combined with CQ phosphate in the treatment of coronavirus pneumonia is being investigated in the ChiCTR2000030987 clinical trial. In ChiCTR2000029609, the efficacy of CQ alone, LPV/RTV alone and CQ plus LPV/RTV is being investigated in mild and severe patients [103]. Two or more drug combinations with CQ are also being investigated in other countries. For example, in THDMS-COVID19, oseltamivir 300 mg per day plus CQ 1000 mg per day or darunavir 400 mg every 8 h RTV 2.5 mg/kg plus oseltamivir 4–6 mg/kg plus CQ 500 mg are being studied [105].

The dosing system and duration of treatment are still varied between different studies. For example, in ChiCTR2000029992, the dosing of CQ is 1.0 g x 2 days for the first dose, 0.5 g x 12 days from the third day, for HCQ is 0.2 g twice daily x 14 days. In ChiCTR2000029975, 150 mg CQ phosphate is dissolved in 5 ml of normal saline, twice daily, and inhaled by atomization for 1 week. The dosing in ChiCTR2000029899 and ChiCTR2000029898 is for HCQ: day 1: first dose: 6 tablets (0.1 g/table, second dose: six tablets (0.1g/tablet) after 6h; day 2–5: two tablets (0.1 g/tablet), twice daily and for CQ day1–3: 500 mg, twice daily. Day 4–5: 250 mg, twice daily. In ChiCTR2000029559, HCQ is given in two doses, and one group received 0.1 oral 2/day, the second group received 0.2 oral 2/day, while the third group is a placebo. In ChiCTR2000029542, CQ is given as 0.5 g every time, twice daily for a 10-day course. More clinical trials are needed to determine the effective dosing regimen as toxicities (cardiomyopathy and retinopathy) are still possible after prolonging and high doses of HCQ [103]. The application of CQ and HCQ for SARS-CoV-2 should also consider contraindications, including patient allergies, glucose-6-phosphate dehydrogenase (G6PD) deficiency, previous history of retinopathy, cardiomyopathy (QT prolongation) or end-stage kidney disease [96].

A recent protocol approved by the office of National Clinical Advisor, acute hospital division, recommended the use of CQ 500 mg twice daily for 10 days for patients diagnosed as mild, moderate and severe cases. Alternatively, HCQ in a regimen of day 1: 400 mg twice daily, then days 2–5, 200 mg twice daily (total duration 5 days). Another alternative is RVD as an intravenous infusion, 200 mg on day 1, then 100 mg on days 2–10 (total duration 10 days) [77]. A novel combination of CQ and azithromycin was used recently in a small French clinical trial [46]. About 36 patients were included, including asymptomatic patients, patients with upper respiratory tract symptoms, and patients with lower respiratory tract symptoms were included. About 20 patients received HCQ sulfate 200 mg, three-times daily for 10 days. Out of the HCQ-treated patients, six patients received azithromycin 500 mg on day 1 and 250 mg for the following 4 days. On day 6, virological clearance was significantly higher in HCQ compared with the control group (70 vs 12.5%). Azithromycin + HCQ group showed 100% virological clearance on day 6. However, this combination is not established yet. The risk of developing severe QT interval prolongation may be a limiting factor. The sample size in this study is small, and more significant studies are needed [99]. A very recent study utilized global affinity purification-mass spectrometry (APMS) analysis to identify the host-pathogen interferences for SARS-CoV-2, further validate the possible efficacies of CQ and azithromycin. The study showed that the SARS-CoV-2 Nsp6 protein interacted with the sigma receptor. The sigma receptors interact with CQ. Azithromycin has also been shown to be an off-target activity to human mitochondria and ribosomes subunits that interacts with the SARS-CoV-2 Nsp8 protein (MRPS27, MRPS5, MRPS25 and MRPS2 ) [106]. Another unusual combination that was proposed recently is nitazoxanide and HCQ. The studies on SARS-CoV-2 showed that the virus interfered with host pathways that are needed to activate the innate immune system of the patient to fight the infection (immune senescence). SARS-CoV-2 blocks the interferon pathway and prevents its upregulation in the host system. Nitazoxanide is antiprotozoal that upregulates the innate antiviral mechanisms and activates interferon pathways in the host [46,107]. Accordingly, the study is proposing that dual use of the HCQ with nitazoxanide would have synergistic effects against SARS-CoV-2 [102]. The proposed dosing system is 400 mg twice a day for 2–3 days (loading) then 200 mg twice a day for 4 days for HCQ and 600 mg –sustained-release (SR) tabs/twice daily of nitazoxanide for 7 days [102].

### Immunomodulators & immunosuppressants

The rationale of using immunosuppressants in the management of SARS-CoV-2-associated pneumonia comes from several reports of cases of hyper inflammation and cytokine storms in the lungs of SARS-CoV-2 patients [108]. These agents have been investigated in previous coronavirus outbreaks and are currently being investigated for SARS-CoV-2 with promising preliminary results for some of these agents [109].

Tocilizumab is a recombinant humanized anti-human IL-6 receptor monoclonal antibody. It acts by inhibiting the binding of IL-6 to IL-6 receptors. It has potent anti-inflammatory effects and already approved for the treatment of RA [110]. Tocilizumab is a biological disease-modifying antirheumatic drug (BoDMARD) and recommended as an alternative to methotrexate or TNF-α antagonists in RA patients [111]. Tocilizumab is well tolerated and associated with an acceptable safety profile [112]. Investigating its role in the management of SARS-CoV-2 associated pneumonia is based on the inflammation and cytokine storm detected in those patients. Key inflammatory mediators including IL-6, IL-2, granulocyte colony-stimulating factor, IFN-γ inducible protein 10 and TNF-α were found to be highly involved in the inflammatory storm inducing severe alveolar destruction and dysfunction in SARS-CoV-2 infections. Elevations in IL-6 levels, in particular, were found as a significant predictor of fatality in patients [113]. Therefore interfering with IL-6 and other mediators may have potential clinical benefits in reversing respiratory dysfunction in those patients. A retrospective study from China hospitals demonstrated the benefits of tocilizumab in addition to the standard therapy of 26 patients with severe and critical care conditions [14,80,98]. Tocilizumab was given at a dose of 400 mg once through an intravenous drip in 18 patients. Another dose of tocilizumab was given for the last three patients due to the fever. Treatment with tocilizumab resulted in a significant relief of fever (100%), lowering of oxygen intake 75% and improvement in respiratory function. Tocilizumab normalized lymphocytes levels 52.6%, significantly decreased the C-reactive protein level 84.2% and improved the CT imaging 90.5%. The results from this study are suggesting tocilizumab as promising therapeutic agent for severe and critical SARS-CoV-2 infections. However, this study is observational study with limited number of patients. According to clinical trials are currently conduced to validate these results [114].

A clinical trial (TOCIVID-19, NCT04317092) supported by the National Cancer Institute was recently registered in Clinicaltrials.gov to investigate the efficacy and tolerability of tocilizumab in the treatment of patients with SARS-CoV-2 infection. Tocilizumab is to be injected as two doses, 8 mg/kg (up to a maximum of 800 mg per dose), with an interval of 12 h [115]. Another clinical study (NCT04315480) has been conducted in Italy is investigating the early administration of single-dose injection of tocilizumab 8 mg/kg in patients with SARS-CoV-2 severe multifocal interstitial pneumonia [116]. Two more Chinese studies are also investigating tocilizumab effectiveness. ChiCTR2000029765 is a multicenter, randomized controlled trial for the efficacy and safety of tocilizumab in the treatment of new coronavirus pneumonia, SARS-CoV-2. The study is being conducted in the First Affiliated Hospital of the University of Science and Technology of China. About 94 patients are receiving standard care therapy, and 94 patients were receiving standard care therapy along with Tocilizumab. ChiCTR2000030894 is another clinical trial investigating the use of FPV combined with tocilizumab versus FPV alone versus tocilizumab alone for treatment of SARS-CoV-2 infection [117]. Sarilumab is another IL-6 blocker that is used in moderate-to-severe RA [118]. It is also being investigated for its effectiveness in the management of SAR-CoV-2 patients in the Chinese clinical trial, NCT04315298 [119]. However, the use of these agents is usually associated with several risks, including the development of secondary infections and severe allergic reactions. Such risks should be considered while using these agents in SARS-CoV-2.

### Immune therapies: bevacizumab as an example

Bevacizumab is another monoclonal antibody (humanized) that blocks the VEGF and interferes with its binding to its receptors. Thus, it inhibits angiogenesis, which is an essential process for the development of new blood vessels to supply cancer cells with oxygen and nutrients [120]. Bevacizumab showed productive anticancer activities against colorectal, kidney, lung, breast and head and neck cancers [111]. Bevacizumab is also being studied as a candidate to manage the SARS-CoV-2 outbreak [23]. An ongoing multicenter clinical trial in Qilu Hospital of Shandong University, China, NCT04305106, is investigating the use of bevacizumab for a severing of critically ill patients with SARS-CoV-2. The study is enrolling 118 patients. Bevacizumab will be administrated in a regimen of 500 mg +0.9% NaCl (100 ml), intravenous drip (not less than 90 min) [121]. NCT04275414 is another similar clinical study that is also established in the same hospital with similar settings on 20 patients. However, the therapeutic benefits of bevacizumab are limited by possible serious events associated with its use. Bevacizumab was reported to result in new-onset hypertension, bleeding, delaying wound healing, thromboembolic events and bowel perforations [111]. These risks should be considered while repurposing the use of this drug for SARS-CoV-2 infection.

Other immunomodulators are being considered, including adalimumab. As mentioned above, TNF-α is an inflammatory mediator that is upregulated in SARS-CoV-2. Adalimumab is a TNF inhibitor and may show effective inhibition of SARS-CoV-2 pneumonia [71].

### Corticosteroids (methylprednisolone in particular)

Previously, corticosteroids have been used to treat the SARS-CoV outbreak with proven efficacy. Accordingly, it is valid to evaluate the role of corticosteroids in the recent SARS-CoV-2 outbreak [122]. Corticosteroids such as methylprednisolone are expected to inhibit inflammatory response that is the main factor inducing lung damage in SARS-CoV-2 infection. On the other hand, corticosteroids may suppress the immune response and delay the viral clearance of SARS-CoV-2 [71]. To investigate their exact effects, a case study by Xu *et al.*, in a 50-year-old patient that was diagnosed with SARS-CoV-2, showed that the administration of methylprednisolone (80 mg twice daily) to the standard care did not improve patients outcomes. The patients' symptoms continued to deteriorate over several days after the medications. Patients developed severe dyspnea and shortness of breath. Oxygen saturation was dropped to less than 60%, and the patient developed cardiac arrest and died [27]. A study included 80 patients with confirmed SARS-CoV-2 infection received treatment of a single antibiotic (moxifloxacin) and RBV antiviral therapy. Only 12 patients received methylprednisolone to improve patients' shortness of breath. The study did not indicate any advantages for the use of methylprednisolone in those patients [28]. Another study was conducted on 46 patients with confirmed SARS-CoV-2 infection who received standard care of oxygen, cough relief, antiviral (LPV/RTV and IFN-α) and nutritional support. Of those, 26 patients additionally received methylprednisolone at a dose of 12 mg/kg/d for 5–7 days via intravenous injection. Three deaths were reported, and two of them received methylprednisolone. Patients received methylprednisolone, showed faster improvement in fever, a shorter period of supplemental oxygen and better absorption of lung focus [122]. Accordingly, methylprednisolone did not improve mortality outcomes but may result in a beneficial impact on clinical symptoms and recovery time. Until the time of this review, the role of corticosteroids as adjunctive treatment in the management of SARS-CoV-2 is still controversial. According to the Centers for Disease Control and Prevention, corticosteroids are not preferred as they may be associated with prolongation of viral replication, as noted in previous MERS-CoV outbreak [68]. Ongoing clinical trials are being conducted at this moment to confirm their role. ChiCTR2000029386 is a randomized clinical trial currently being conducted in China on 48 patients and methylprednisolone to be administrated to half of them like a dose of 1–2 mg/kg/day for 3 days [123]. Other studies also registered for Clinicaltrials.gov and currently in the process of recruiting patients. These studies include NCT04244591 (for critically-ill patients with severe acute respiratory failure (Steroids-SARI) in Beijing, China), NCT04263402 (to compare the efficacy of different hormone doses in the treatment of SARS-CoV-2 severe pneumonia in Wuhan, Hubei, China), and NCT04273321 (to determine efficacy and safety of corticosteroids in SARS-CoV-2 in different areas in China) [124].

### Immune boosters

In contrast to the above reports about the potential benefits of using immunosuppressants and immunomodulators in the management of SARS-CoV-2, a very recent study is suggesting the use of immune booster interventions. The study is proposing the use of individualized interventions to improve the immune response (I4R) approach. Low-level radiation, statins and aspirin are suggested for the treatment of SARS-CoV-2 pneumonia. This proposal stems from previous reports about the effectiveness of these interventions in curing pneumonia in general. The study urges the implantation of clinical studies to investigate these regimens [125].

### Anti-parasitic agent (ivermectin)

Recent *in vitro* reports demonstrated the potential efficacy of ivermectin against SAR-COV-2. 5000-fold reduced the viral load (in 2-h post-infection with SARS-CoV-2 Vero-hSLAM cells) after 48 h of single ivermectin treatment (5 μM) [126]. An observational study showed the survival benefits of using single ivermectin dose (150 mg/kg) after initiating mechanical ventilation in critically ill SAR-COV-2 patients. Patients who received ivermectin showed better survival, shorter hospital stay and intensive care unit length [127]. A recent study from Italy is suggesting a synergistic effect of combining hydroxychloroquine with ivermectin against SAR-COV-2. The study is hypothesizing that the two drugs have no serious interactions and can be safely studied against SAR-COV-2 [128].

## Proposed potential therapies (not in clinical trials yet)

The discovery and development of new molecules against SARS-CoV-2 infection need time [129]. Besides, *in silico* discovery of molecules is relatively a slow process as these molecules need to be experimentally tested. In this section, we summarize the potential therapies as suggested and proposed by several research groups and pharmaceutical companies from the beginning of SARS-CoV-2 infection. The selection of these potential therapies was based on their role in similar viral infections on the corresponding targets.

### Anti-inflammatory therapies

Inflammation is a response of the immune system during the earliest stages of viral infections [130]. As a result, the anti-inflammatory drug is a choice for viral infections, including SARS-CoV-2 infection. Few studies discussed anti-inflammatory therapies as an option to reduce the symptoms of SARS-CoV-2 infection with baricitinib, and melatonin being the most related [131].

Baricitinib, a drug approved for RA, was identified by *in silico* screening for anti-inflammatory drugs that could be potential therapies for SARS-CoV-2 infection [132]. SAR-COV-2 enters lung cells by ACE2-mediated invasion. AP2-associated protein kinase 1 (AAK1) is one of the regulators for this invasion. Few approved drugs with high affinity to inhibit AAK-1 enzymes such as fedratinib and sunitinib were *in silico* screened with the only barcitinib showing an inhibitory effect on the enzyme janus kinase (JAK), another regulator for the invasion. Stebbing *et al.* have suggested that baricitinib could be trialed for its anti-inflammatory and entry inhibition effects [132]. Moreover, the patent application reveals the preparation of baricitinib by Incyte Corporation pharmaceutical company [131].

Melatonin is another drug that has been proposed as a potential anti-inflammatory drug to relieve the symptoms of SARS-CoV-2 infection. Viral infections cause injury of the immune system that is commonly associated with oxidative stress and damage of organs [133]. Melatonin is an old biogenic amine for treating sleep disturbances and circadian rhythm. It has an antioxidant effect and has been previously encouraged to be used for Ebola viral infection [134]. As suggested by Cheng *et al.*, melatonin could reduce the clinical symptoms of SARS-CoV-2 infection and prolong the survival time for the patient.

### Angiotensin receptor blocking therapies

As mentioned previously, SARS-CoV-2 gets into the pulmonary cells after binding to the ACE2 receptor domain [135]. However, due to the limited number of studies, it is still unclear how ACE2 is modified in SARS-CoV-2 infection [32,135]. Kruse has suggested that therapies that block the ACE2 receptor domain could be studied for their potential effectiveness against SARS-CoV-2 infection [136]. For example, using the small receptor-binding domain (RBD) from the vital domain of the SARS S protein that has shown to bind to ACE2 [136,137] receptor. A second option is to administer an antibody that binds to ACE2, which could prevent SARS-CoV-2 particles from binding to the ACE2 receptor. A third potential option that has been suggested by Zhang and Liu is using compounds that have shown to inhibit ACE2 enzyme such as emodin and promazine [90]. Emodin is a natural anthraquinone compound that is derived from the commonly used Chinese medicinal herbs, such as the genus *Rheum* and *Polygonum* [138]. Emodin was found to block the binding of SARS-CoV S protein with the enzyme ACE2 [139], while promazine is an old anti-psychotic drug with structural similarity to emodin. Promazine has shown inhibitory signs for the replication of SARS-CoV. From these findings, Ho *et al.* have suggested that emodin or promazine could be considered as potential therapeutics for SARS-CoV-2 infection.

### Protease inhibitors

Having coronaviral principal protease (3CLpro) as the critical enzyme for SARS-CoV-2 replication and serine protease (TMPRSS211) for S protein priming has made these proteases attractive targets [140]. Thus, the existing protease inhibitors could be potential drug candidates targeting these enzymes [140]. Few antivirals were mentioned previously as having a 3CLpro inhibitory effect such as LPV and RTV.

Carboxamide derivatives have been shown to have an antiviral inhibitory effect targeting 3CLpro [140] and are undergoing studies in research and development [32], as reported by Liu *et al.* From these carboxamide derivatives, ML188 analog has IC_50_ of 1.5 μM, and ML300 analog has IC_50_ of 6.2 μM. Benzenepropanamide derivatives have also shown a disruption effect for the function of proteases 3CLpro and Plpro in SARS-CoV infection [141]. These candidates are also undergoing studies in research and development, as reported by Liu *et al.* Few peptidomimetic compounds and GC376, a previously known protease inhibitor, have been reported to inhibit protease 3Clpro [141,142]. α-ketoamide inhibitors were previously synthesized and tested against MERS-CoV infection by Liu research group. α-ketoamide derivatives have shown picomolar activity against protease 3CLpro of MERS-CoV. Hilgenfeld *et al.* have recently reported the x-ray structures of SARS-CoV3 3Clpro and its complex with an α-ketoamide inhibitor and considered α-ketoamide inhibitors as promising agents for SARS-CoV-2 infection [140].

### Traditional Chinese medicines therapy

Traditional Chinese medicines (TCMs) include different types of natural products, with each relating to a group of diseases [143]. TCMs that have shown activity against viral infections, specifically lung infections, could be potential therapies for relieving the symptoms of SARS-CoV-2 infection, as proposed in the literature by different research groups [43,139,144]. It worth mentioning herein that TCMs have been used in the control of several epidemics and pandemic diseases over thousands of years, which shows their effectiveness [143,145,146]. Moreover, TCMs were included in the guideline for the diagnosis and treatment of COVID-19 by the National Health Commission of the People's Republic of China [145]. Results have shown that the symptoms of COVID-19 were shortened in 60,107 confirmed cases. However, more clinical studies are needed that require time.

Jian-Ping *et al.* have done a study based on historical records on the prevention and treatment of infections using TCM, TCM prevention programs issued by Chinese health authorities and databases, and preliminary literature results about using TCM in other respiratory viral infections [147]. Based on the results, Jian-Ping *et al.* have confirmed the use of TCM as a preventive therapy for SARS-CoV-2 infection. Another trial study that is under process is also using TCM. In this study, interventional subjects will receive triple therapy includes oxidative therapy, an antiviral and TCM [143]. Zhang *et al.* have concluded that TCMs could contain direct constituents for the treatment of COVID-19 [139]. In this study, it has been identified as different TCMs that have been previously used in treating respiratory viral infections by an *in silico* screening.

### Therapies in research & development

In addition to current and potential therapies, several others are currently in the research and development process. Several pharmaceutical companies enter the race to find a treatment for SARS-CoV-2 infection, relieve its symptoms or to reduce the risk of its complications (Table 2) [148].

**Table 2.** Therapies in research and development as potential therapies for SARS-CoV-2 infection.

Therapies	Type	Pharmaceutical company	Website
TJM2	Neutralizing antibody	I-Mab Biopharma	http://www.i-mabbiopharma.com/en/article-491.aspx
AT-100	Human recombinant protein	Airway Therapeutics	https://www.airwaytherapeutics.com/at-100/
TZLS-501	Human monoclonal antibody (mAb)	Tiziana Life Sciences	https://www.tizianalifesciences.com/our-drugs/anti-il-6r/
OYA1	Antiviral	OyaGeninc	http://www.oyageninc.com/wordpress/drugs
NP-120	Antifibrotic	Algernon Pharmaceuticals	https://algernonpharmaceuticals.com/?s=NP-120
APN01	Recombinant human angiotensin-converting enzyme 2 (rhACE2)	Apeiron Biologics	https://www.apeiron-biologics.com/project-overview/#APN01
Brilacidin	Antibacterial, anti-inflammatory, and immune modulator	Innovation Pharmaceuticals	http://www.ipharminc.com/brilacidin-1?rq=Brilacidin
Leronlimab	Monoclonal antibody	CytoDyn	https://www.cytodyn.com/our-science
REGN3048 and REGN3051	Combination of neutralizing monoclonal antibodies	Regeneron	https://www.regeneron.com/search-regeneron?query=REGN3048
SNG001	Interferon-β	Synairgen Research	https://www.synairgen.com/covid-19/
Nanobody		Beroni Group	https://www.beronigroup.com/2020/03/13/beroni-group-advances-research-and-development-of-medical-solution-for-coronavirus-covid-19/
Galidesivi**r**	Antiviral	Biocryst	https://www.biocryst.com/our-program/galidesivir/
CYNK-001	Natural killer (NK) cell therapy	Celularity and Sorrento Therapeutics	https://www.celularity.com/
Remestemcel-L	Allogeneic mesenchymal stem cell (MSC) product candidate	Mesoblast	http://investorsmedia.mesoblast.com/static-files/c1428818-0b9f-44f9-bb4f-79ad518002cc
MAN-01	Antiglaucoma	Q Biomed and Mannin Research	https://www.sec.gov/Archives/edgar/data/1596062/000110465920027119/tm205231d1_10k.htm
Opaganib/RHB-107	Anticancer and anti-inflammatory/potential for use in multiple oncology gastrointestinal and indications	RedHill Biopharma	https://www.redhillbio.com/RedHill/Templates/showpage.asp?DBID=1&LNGID=1&TMID=178&FID=2432&PID=0&IID=13253
Antibody cocktail therapy	–	Regeneron Pharmaceuticals	https://investor.regeneron.com/news-releases/news-release-details/regeneron-announces-important-advances-novel-covid-19-antibody

## Conclusion

The review offers reported information on the global research and advancement of potential and current therapeutic agents and vaccines under development relevant for SARS-CoV-2 focused on the complete, up-to-date generic drug product selection. This describes morphology, physiology and pathogenesis of SARS-CoV-2 and focuses primarily on antivirals, antimalarials and immunotherapeutics aimed at diverse molecular associations linked to infection and replication. This review focuses primarily on agents reported to be beneficial against certain RNA viruses such as SARS-CoV, MERS-CoV, influenza, Ebola and counter inflammatory medications, as well as product reuse attempts. There is a broad range of products available in recombinant biotechnology to generate antibodies as well as in cytokines targeted at the production and transmission of virus genes and cellular receptors. A significant attempt will be made to produce successful drugs and vaccinations against current and possible future SARS-CoV-2 infections and other potentially pathogenic virus outbreaks to minimize the tremendous effect on human life. The epidemic of COVID-19 underlines further the importance of developing a relatively broad spectrum of antiviral drugs and the significance of implementing innovative strategies like artificial intelligence to expedite therapeutics development, considering the cost and effort involved in developing clinical drugs. As highlighted in this review, the abundance of publications and the fast publishing race associated with the virus SARS-CoV-2 outbreak suggests a concentrated initiative on the part of research organizations and the pharmaceutical industry both concerning molecular mechanisms and concerning therapeutic pathways used for current and potential coronavirus outbreaks.

## Future perspective

This overview of the current COVI19 global interest offers a summary of the existing state of the art concerning effects on public health and safety, pathophysiology and clinical symptoms of the pandemic infection, diagnostics tools available, patient management and global response from enforced curfew to total lockdown that we are witnessing around the globe. Only when the pandemic ceases will we evaluate the health, social and economic consequences of this catastrophic event and, therefore, can draw insights from any possible potential epidemics for future cases, particularly in public and global safety. The proverb of “*an ounce of prevention is worth a pound of cure*” remains the best for the prevention of COVID-19 spread.

**Executive summary**

- The pandemic of COVID-19 has very significant medical, economic and social consequences.
- Government agencies respond differently in various forms, but there are still extensive limitations on people's gatherings, public meetings, football matches, sports and social/leisure events all have been suspended globally.
- Care for COVID-19 patients is close to the provided therapy for many other pneumonia-causing viruses or infections. This primarily consists of preventive treatment and supplementation with oxygen if required.
- The active research on COVID-19 treatments, the intensity and amount of clinical trials underway demonstrate the necessity and willingness to deliver high-quality data even in a disease outbreak. To date, no successful treatments have been identified yet.
- ClinicalTrials.gov has 291 trails specific to COVID-19 of 351 clinical studies on 13 April 2020.
- Potential investigated therapies and potential examined vaccinations of COVID-19 are currently at the forefront of research and global priority.

## References

[1] Jeevanandam J, Pal K, Danquah MK Virus-like nanoparticles as a novel delivery tool in gene therapy. Biochimie 157, 38–47 (2019). 30408502 10.1016/j.biochi.2018.11.001

[2] Aljabali AA, Sainsbury F, Lomonossoff GP, Evans DJ Cowpea mosaic virus unmodified empty virus-like particles loaded with metal and metal oxide. Small. 6(7), 818–821 (2010). 20213652 10.1002/smll.200902135

[3] Cascella M, Rajnik M, Cuomo A, Dulebohn SC, Di Napoli R Features, evaluation and treatment coronavirus (COVID-19). StatPearls, Treasure Island (FL) (2020). 32150360

[4] Chen WH, Strych U, Hotez PJ, Bottazzi ME The SARS-CoV-2 vaccine pipeline: an overview. Curr Trop Med Rep. 1–4 (2020). 10.1007/s40475-020-00201-6 PMC7094941 32219057

[5] Ferretti L, Wymant C, Kendall M Quantifying SARS-CoV-2 transmission suggests epidemic control with digital contact tracing. Science (2020). 10.1126/science.abb6936 PMC7164555 32234805

[6] Wrapp D, Wang N, Corbett KS Cryo-EM structure of the 2019-nCoV spike in the prefusion conformation. Science 367(6483), 1260–1263 (2020). 32075877 10.1126/science.abb2507 PMC7164637

[7] Woo PC, Huang Y, Lau SK, Yuen K-Y Coronavirus genomics and bioinformatics analysis. Vruses 2(8), 1804–1820 (2010). 10.3390/v2081803 PMC3185738 21994708

[8] Drexler JF, Gloza-Rausch F, Glende J Genomic characterization of severe acute respiratory syndrome-related coronavirus in European bats and classification of coronaviruses based on partial RNA-dependent RNA polymerase gene sequences. J. Virol. 84(21), 11336–11349 (2010). 20686038 10.1128/JVI.00650-10 PMC2953168

[9] Yin Y, Wunderink RG MERS, SARS and other coronaviruses as causes of pneumonia. Respirology 23(2), 130–137 (2018). 29052924 10.1111/resp.13196 PMC7169239

[10] Peiris J, Lai S, Poon L Coronavirus as a possible cause of severe acute respiratory syndrome. Lancet 361(9366), 1319–1325 (2003). 12711465 10.1016/S0140-6736(03)13077-2 PMC7112372

[11] Zaki AM, Van Boheemen S, Bestebroer TM, Osterhaus AD, Fouchier RA Isolation of a novel coronavirus from a man with pneumonia in Saudi Arabia. N. Engl. J. Med. 367(19), 1814–1820 (2012). 23075143 10.1056/NEJMoa1211721

[12] Imai N, Dorigatti I, Cori A, Donnelly C, Riley S, Ferguson NM Report 2: Estimating the potential total number of novel Coronavirus cases in Wuhan City, China. Imperial College London (2020).

[13] Din MaU, Boppana LKT An update on the 2019-nCoV outbreak. Am. J. Infect. Control (2020) (Epub ahead of print). 10.1016/j.ajic.2020.01.023 PMC7102631 32171622

[14] Liu F, Xu A, Zhang Y Patients of COVID-19 may benefit from sustained lopinavir-combined regimen and the increase of eosinophil may predict the outcome of COVID-19 progression. Int. J. Infect. Dis. (2020). 10.1016/j.ijid.2020.03.013 PMC7193136 32173576

[15] RVB. Division of viral diseases. Real-time rt-pcr panel for detection 2019- novel coronavirus. (2020). www.who.int/docs/default-source/coronaviruse/uscdcrt-pcr-panel-for-detectioninstructions.pdf?sfvrsn=3aa07934_2

[16] Andersen KG, Rambaut A, Lipkin WI, Holmes EC, Garry RF The proximal origin of SARS-CoV-2. Nat. Med. 26(4), 1–3 (2020). 32284615 10.1038/s41591-020-0820-9 PMC7095063

[17] Fan J, Liu X, Pan W, Douglas M, Bao S Epidemiology of 2019 Novel Coronavirus Disease-19 in Gansu Province, China, 2020. Emerg. Infect. Dis. 26(6), (2020). 10.3201/eid2606.200251 PMC7258465 32168465

[18] Ahn DG, Shin HJ, Kim MH Current Status of Epidemiology, Diagnosis, Therapeutics, and Vaccines for Novel Coronavirus Disease 2019 (COVID-19). J. Microbiol. Biotechnol. 30(3), 313–324 (2020). 32238757 10.4014/jmb.2003.03011 PMC9728410

[19] Chen Z, Zhang W, Lu Y From SARS-CoV to Wuhan 2019-nCoV Outbreak: Similarity of Early Epidemic and Prediction of Future Trends. Cell-Host-Microbe-D-20-00063 (2020). 10.2139/ssrn.3528722

[20] Hoffmann M, Kleine-Weber H, Schroeder S SARS-CoV-2 cell entry depends on ACE2 and TMPRSS2 and is blocked by a clinically proven protease inhibitor. Cell 181(2), 271–280.e8 (2020). 32142651 10.1016/j.cell.2020.02.052 PMC7102627

[21] Ziebuhr J, Snijder EJ, Gorbalenya AE Virus-encoded proteinases and proteolytic processing in the Nidovirales. J. Gen. Virol. 81(4), 853–879 (2000). 10725411 10.1099/0022-1317-81-4-853

[22] Báez-Santos YM, John SES, Mesecar AD The SARS-coronavirus papain-like protease: structure, function and inhibition by designed antiviral compounds. Antiviral Res. 115, 21–38 (2015). 25554382 10.1016/j.antiviral.2014.12.015 PMC5896749

[23] Zhao Y, Wei Y, Shen S, Zhang M, Chen F Appealing for efficient, well organized clinical trials on COVID-19. MedRxiv. 20031476, (2020) (Epub ahead of print). 10.21037/atm-20-2429 PMC7290614 32566569

[24] Wan Y, Shang J, Graham R, Baric RS, Li F Receptor recognition by the novel coronavirus from Wuhan: an analysis based on decade-long structural studies of SARS coronavirus. J. Virol. 94(7), (2020). 10.1128/JVI.00127-20 PMC7081895 31996437

[25] Peng Z, Xing-Lou Y, Xian-Guang W A pneumonia outbreak associated with a new coronavirus of probable bat origin. [J/OL]. Nature 579, 270–273 (2020). 32015507 10.1038/s41586-020-2012-7 PMC7095418

[26] Wu F, Zhao S, Yu B A new coronavirus associated with human respiratory disease in China. Nature 579(7798), 265–269 (2020). 32015508 10.1038/s41586-020-2008-3 PMC7094943

[27] Xu Z, Shi L, Wang Y Pathological findings of COVID-19 associated with acute respiratory distress syndrome. Lancet Respir. Med. 8(4), 420–422 (2020). . 32085846 10.1016/S2213-2600(20)30076-X PMC7164771

[28] Wu J, Liu J, Zhao X Clinical characteristics of imported cases of COVID-19 in Jiangsu province: a multicenter descriptive study. Clin. Infect. Dis. pii: ciaa199 (2020) (Epub ahead of print). 10.1093/cid/ciaa199 PMC7108195 32109279

[29] WHO. Coronavirus disease (COVID-2019) situation reports. (2020). www.who.int/emergencies/diseases/novel-coronavirus-2019/situation-reports

[30] Al Johani S, Hajeer AH MERS-CoV diagnosis: an update. J. Infect. Public Health 9(3), 216–219 (2016). 27106390 10.1016/j.jiph.2016.04.005 PMC7102781

[31] WHO. Novel coronavirus (2019-ncov) technical guidance: laboratory testing for 2019-ncov in humans. (2020). www.who.int/emergencies/diseases/novel-coronavirus-2019/technical-guidance/laboratory-guidance

[32] Liu C, Zhou Q, Li Y Research and Development on Therapeutic Agents and Vaccines for COVID-19 and Related Human Coronavirus Diseases. ACS Central Science 6(3), 315–331 (2020). 32226821 10.1021/acscentsci.0c00272 PMC10467574

[33] Corman VM, Landt O, Kaiser M Detection of 2019 novel coronavirus (2019-nCoV) by real-time RT-PCR. Eurosurveillance 25(3), (2020). 10.2807/1560-7917.ES.2020.25.3.2000045 PMC6988269 31992387

[34] Kim M-N, Ko YJ, Seong M-W, Kim J-S, Shin B-M, Sung H Analytical and clinical validation of six commercial Middle East Respiratory Syndrome coronavirus RNA detection kits based on real-time reverse-transcription PCR. Ann. Lab. Med. 36(5), 450–456 (2016). 27374710 10.3343/alm.2016.36.5.450 PMC4940488

[35] Shirato K, Yano T, Senba S Detection of Middle East respiratory syndrome coronavirus using reverse transcription loop-mediated isothermal amplification (RT-LAMP). Virol. J. 11(1), 139 (2014). 25103205 10.1186/1743-422X-11-139 PMC4132226

[36] Hashemzadeh MS, Rasouli R, Zahraei B Development of dual taqman based one-step rrt-pcr assay panel for rapid and accurate diagnostic test of MERS-CoV: a novel human coronavirus, ahead of Hajj pilgrimage. Iran Red. Crescent. Med. J. 18(11), e23874 (2016). 28191331 10.5812/ircmj.23874 PMC5292137

[37] Lau SK, Che X-Y, Woo PC SARS coronavirus detection methods. Emerg. Infect. Dis. 11(7), 1108 (2005). 16022791 10.3201/eid1107.041045 PMC3371792

[38] Lau LT, Fung Y-WW, Wong FP-F A real-time PCR for SARS-coronavirus incorporating target gene pre-amplification. Biochem. Biophys. Res. Commun. 312(4), 1290–1296 (2003). 14652014 10.1016/j.bbrc.2003.11.064 PMC7111096

[39] Jiang SS, Chen T-C, Yang J-Y Sensitive and quantitative detection of severe acute respiratory syndrome coronavirus infection by real-time nested polymerase chain reaction. Clin. Infect. Dis. 38(2), 293–296 (2004). 14699465 10.1086/380841 PMC7107825

[40] He Q, Chong KH, Chng HH Development of a Western blot assay for detection of antibodies against coronavirus causing severe acute respiratory syndrome. Clin. Diagn. Lab. Immunol. 11(2), 417–422 (2004). 15013997 10.1128/CDLI.11.2.417-422.2004 PMC371214

[41] Hui RK, Zeng F, Chan CM, Yuen K, Peiris JS, Leung FC Reverse transcriptase PCR diagnostic assay for the coronavirus associated with severe acute respiratory syndrome. J. Clin. Microbiol. 42(5), 1994–1999 (2004). 15131160 10.1128/JCM.42.5.1994-1999.2004 PMC404607

[42] Wu JT, Leung K, Leung GM Nowcasting and forecasting the potential domestic and international spread of the 2019-nCoV outbreak originating in Wuhan, China: a modelling study. Lancet 395(10225), 689–697 (2020). 32014114 10.1016/S0140-6736(20)30260-9 PMC7159271

[43] Pang J, Wang MX, Ang IYH Potential rapid diagnostics, vaccine and therapeutics for 2019 novel Coronavirus (2019-ncoV): a systematic review. J. Clin. Med. 9(3), 623 (2020). 10.3390/jcm9030623 PMC7141113 32110875

[44] Detection of 2019 novel coronavirus (2019-ncov) in suspected human cases by rt-pcr lks faculty of medicine school of public health 2020). www.who.int/docs/default-source/coronaviruse/peiris-protocol-16-1-20.pdf?sfvrsn=af1aac73_4

[45] Kelly-Cirino C, Mazzola LT, Chua A, Oxenford CJ, Van Kerkhove MD An updated roadmap for MERS-CoV research and product development: focus on diagnostics. BMJ Glob. Health 4(Suppl. 2), e001105 (2019). 10.1136/bmjgh-2018-001105 PMC6361340 30815285

[46] Nguyen T, Duong Bang D, Wolff A 2019 Novel Coronavirus Disease (COVID-19): Paving the Road for Rapid Detection and Point-of-Care Diagnostics. Micromachines 11(3), 306 (2020). 10.3390/mi11030306 PMC7142866 32183357

[47] Xiong X, Tortorici MA, Snijder J Glycan shield and fusion activation of a deltacoronavirus spike glycoprotein fine-tuned for enteric Infections. J. Virol. 92(4), (2018). 10.1128/JVI.01628-17 PMC5790929 29093093

[48] Song W, Gui M, Wang X, Xiang Y Cryo-EM structure of the SARS coronavirus spike glycoprotein in complex with its host cell receptor ACE2. PLoS Pathog. 14(8), e1007236 (2018). 30102747 10.1371/journal.ppat.1007236 PMC6107290

[49] Vankadari N, Wilce JA Emerging WuHan (COVID-19) coronavirus: glycan shield and structure prediction of spike glycoprotein and its interaction with human CD26. Emerg. Microbes Infect. 9(1), 601–604 (2020). 32178593 10.1080/22221751.2020.1739565 PMC7103712

[50] Bgi develops real-time fluorescent rt-pcr kit for detecting the 2019 novel coronavirus 2020). www.bgi.com/global/company/news/bgi-develops-real-time-dna-based-kit-for-detecting-the-2019-novel-coronavirus/

[51] Lin J, Zhang J-S, Su N Safety and immunogenicity from a phase I trial of inactivated severe acute respiratory syndrome coronavirus vaccine. Antivir. Ther. 12(7), 1107 (2007). 18018769

[52] Beigel JH, Voell J, Kumar P Safety and tolerability of a novel, polyclonal human anti-MERS coronavirus antibody produced from transchromosomic cattle: a phase 1 randomised, double-blind, single-dose-escalation study. Lancet Infect. Dis. 18(4), 410–418 (2018). 29329957 10.1016/S1473-3099(18)30002-1 PMC5871563

[53] Modjarrad K, Roberts CC, Mills KT Safety and immunogenicity of an anti-Middle East respiratory syndrome coronavirus DNA vaccine: a phase 1, open-label, single-arm, dose-escalation trial. Lancet Infect. Dis. 19(9), 1013–1022 (2019). 31351922 10.1016/S1473-3099(19)30266-X PMC7185789

[54] Evaluate the safety, tolerability and immunogenicity study of gls-5300 in healthy volunteers. (2020). https://clinicaltrials.gov/ct2/show/study/NCT03721718?term=vaccine&cond=Mers+CoV&draw=2&rank=7

[55] Efficacy and safety of hydroxychloroquine for treatment of pneumonia caused by 2019-ncov (hc-ncov) 2020). https://clinicaltrials.gov/ct2/show/NCT04261517

[56] Ahmed SF, Quadeer AA, Mckay MR Preliminary identification of potential vaccine targets for the COVID-19 Coronavirus (SARS-CoV-2) Based on SARS-CoV Immunological Studies. Viruses 12(3), (2020). 10.3390/v12030254 PMC7150947 32106567

[57] Chen WH, Hotez PJ, Bottazzi ME Potential for developing a SARS-CoV receptor-binding domain (RBD) recombinant protein as a heterologous human vaccine against coronavirus infectious disease (COVID)-19. Hum. Vaccin. Immunother. 1–4 (2020). 10.1080/21645515.2020.1740560 PMC7482854 32298218

[58] Cohen J Vaccine designers take first shots at COVID-19. Science 368(6486), 14–16 (2020). 32241928 10.1126/science.368.6486.14

[59] Enayatkhani M, Hasaniazad M, Faezi S Reverse vaccinology approach to design a novel multi-epitope vaccine candidate against COVID-19: an *in silico* study. J. Biomol. Struct. Dyn., 1–19 (2020). 10.1080/07391102.2020.1756411 PMC7196925 32295479

[60] Peeples L News Feature: avoiding pitfalls in the pursuit of a COVID-19 vaccine. Proc. Natl Acad. Sci. USA 117(15), 8218–8221 (2020). 32229574 10.1073/pnas.2005456117 PMC7165470

[61] Rosales-Mendoza S, Marquez-Escobar VA, Gonzalez-Ortega O, Nieto-Gomez R, Arevalo-Villalobos JI What does plant-based vaccine technology offer to the fight against COVID-19? Vaccines 8(2), (2020). 10.3390/vaccines8020183 PMC7349371 32295153

[62] Shoenfeld Y Corona (COVID-19) time musings: our involvement in COVID-19 pathogenesis, diagnosis, treatment and vaccine planning. Autoimmun. Rev. (2020) (Epub ahead of print). 10.1016/j.autrev.2020.102538 PMC7131471 32268212

[63] Thanh Le T, Andreadakis Z, Kumar A The COVID-19 vaccine development landscape. Nat. Rev. Drug Discov. (2020) (Epub ahead of print). 10.1038/d41573-020-00073-5 32273591

[64] Benvenuto D, Giovanetti M, Ciccozzi A, Spoto S, Angeletti S, Ciccozzi M The 2019-new coronavirus epidemic: evidence for virus evolution. J. Med. Virol. 92(4), 455–459 (2020). 31994738 10.1002/jmv.25688 PMC7166400

[65] Rosa SGV, Santos WC Clinical trials on drug repositioning for COVID-19 treatment. (2020) 44, e40 10.26633/RPSP.2020.40 PMC7105280 32256547

[66] Choudhary S, Malik YS, Tomar S, Tomar S Identification of SARS-CoV-2 cell entry inhibitors by drug repurposing using *in silico* structure-based virtual screening approach (2020). 10.3389/fimmu.2020.01664 PMC7365927 32754161

[67] Farag A, Wang P, Ahmed M, Sadek H Identification of FDA approved drugs targeting COVID-19 virus by structure-based drug repositioning (2020). .

[68] Center for Disease Control and Prevention. Interim clinical guidance for management of patients with confirmed coronavirus disease (COVID-19). (2020). www.cdc.gov/coronavirus/2019-ncov/hcp/clinical-guidance-management-patients.html

[69] World Health Organization. Clinical management of severe acute respiratory infection (SARI) when COVID-19 disease is suspected. March 2020 (2020). www.who.int/publications-detail/clinical-management-of-severe-acute-respiratory-infection-when-novel-coronavirus-(ncov)-infection-is-suspected

[70] Nicholas J, Beeching TEF, Fowler Robert BMJ Best Practice: coronavirus disease 2019 (COVID-19) (2020). https://bestpractice.bmj.com/topics/en-gb/3000168

[71] Favalli EG, Ingegnoli F, De Lucia O, Cincinelli G, Cimaz R, Caporali R COVID-19 infection and rheumatoid arthritis: faraway, so close! Autoimmun. Rev. 19(5), 102523 (2020). 10.1016/j.autrev.2020.102523 32205186 PMC7102591

[72] Cao B, Wang Y, Wen D A trial of lopinavir–ritonavir in adults hospitalized with severe Covid-19. N. Engl. J. Med. (2020) (Epub ahead of print). 10.1056/NEJMoa2001282 PMC7121492 32187464

[73] Cai Q, Yang M, Liu D Experimental Treatment with Favipiravir for COVID-19: An Open-Label Control Study. Engineering (2020). 10.1016/j.eng.2020.03.007 PMC7185795 32346491

[74] Holshue ML, Debolt C, Lindquist S First case of 2019 novel coronavirus in the United States. N. Engl. J. Med. 382(10), 929–936 (2020). 32004427 10.1056/NEJMoa2001191 PMC7092802

[75] Chen N, Zhou M, Dong X Epidemiological and clinical characteristics of 99 cases of 2019 novel coronavirus pneumonia in Wuhan, China: a descriptive study. Lancet 395(10223), 507–513 (2020). 32007143 10.1016/S0140-6736(20)30211-7 PMC7135076

[76] Wang D, Hu B, Hu C Clinical characteristics of 138 hospitalized patients with 2019 novel coronavirus – infected pneumonia in Wuhan, China. JAMA 323(11), 1061–1069 (2020). 10.1001/jama.2020.1585 PMC7042881 32031570

[77] Bergin C, Philbin M, Gilvarry P, O'connor M, King F Specific Antiviral Therapy in the Clinical Management of Acute Respiratory Infection with SARS-CoV-2 (COVID-19). ww.hse.ie/eng/about/who/acute-hospitals-division/drugs-management-programme/guidelines/specific-antiviral-therapy-in-the-clinical-management-of-acute-respiratory-infection-with-sars-cov-2-covid-19.pdf

[78] Dong L, Hu S, Gao J Discovering drugs to treat coronavirus disease 2019 (COVID-19). Search Results Featured snippet from the web Drug Discov Ther. 14(1), 58–60 (2020). 10.5582/ddt.2020.01012 32147628

[79] Chang Y-C, Tung Y-A, Lee K-H *et* *al.* Potential therapeutic agents for COVID-19 based on the analysis of protease and RNA polymerase docking. 2020020242 (2020). doi: 10.20944/preprints202002.0242.v1

[80] Cao B, Wang Y, Wen D A trial of lopinavir-ritonavir in adults hospitalized with severe Covid-19. N. Engl. J. Med. (2020) (Epub ahead of print). 10.1056/NEJMoa2001282 PMC7121492 32187464

[81] Feikin DR, Schuchat A, Kolczak M Mortality from invasive pneumococcal pneumonia in the era of antibiotic resistance, 1995–1997. Am. J. Public Health 90(2), 223 (2000). 10667183 10.2105/ajph.90.2.223 PMC1446155

[82] Zheng XW, Tao G, Zhang YW, Yang GN, Huang P Drug interaction monitoring of lopinavir / ritonavir in COVID-19 patients with cancer. Zhonghua Nei Ke Za Zhi 59(8(4)), 420–422 (2020). 10.3760/cma.j.cn112138-20200219-00097 32114746

[83] Clinicaltrials.Gov. Clinical trials on Favipiravir role in SARS-COV-2 infection. (2020). https://clinicaltrials.gov/ct2/results?cond=covid+19&term=Favipiravir+&cntry=&state=&city=&dist=

[84] Wang M, Cao R, Zhang L Remdesivir and chloroquine effectively inhibit the recently emerged novel coronavirus (2019-nCoV) *in vitro*. Cell Res. 30(3), 269–271 (2020). 32020029 10.1038/s41422-020-0282-0 PMC7054408

[85] Clinicaltrials.Gov. Study to Evaluate the Safety and Antiviral Activity of Remdesivir (GS-5734™) in Participants With Severe Coronavirus Disease (COVID-19). (2020). https://clinicaltrials.gov/ct2/show/NCT04292899

[86] Barlow A, Landolf KM, Barlow B Review of Emerging Pharmacotherapy for the Treatment of Coronavirus Disease 2019. Pharmacotherapy (2020) (Epub ahead of print). 10.1002/phar.2398 PMC7262196 32259313

[87] Hillaker E, Belfer JJ, Bondici A, Murad H, Dumkow LE Delayed Initiation of Remdesivir in a COVID-19 Positive Patient. Pharmacotherapy (2020) (Epub ahead of print). 10.1002/phar.2403 PMC7262085 32281114

[88] Health NIO NIH clinical trial of remdesivir to treat COVID-19 begins. (2020). www.nih.gov/news-events/news-releases/nih-clinical-trial-remdesivir-treat-covid-19-begins

[89] Elfiky AA Anti-HCV, nucleotide inhibitors, repurposing against COVID-19. Life Sci. 248, 117477 (2020). 32119961 10.1016/j.lfs.2020.117477 PMC7089605

[90] Zhang L, Liu Y Potential interventions for novel coronavirus in China: a systematic review. J. Med. Virol. 92(5), 479–490 (2020). 32052466 10.1002/jmv.25707 PMC7166986

[91] Clinicaltrials.Gov. An Open-label Randomized Controlled Trial on Lopinavir/ Ritonavir, Ribavirin and Interferon Beta 1b Combination Versus Lopinavir/ Ritonavir Alone, as Treatment for 2019 Novel Coronavirus Infection. (2020). https://clinicaltrials.gov/ct2/show/NCT04276688

[92] Xu K, Cai H, Shen Y Management of corona virus disease-19 (COVID-19): the Zhejiang experience. Zhejiang da xue xue bao. Yi xue ban=Journal of Zhejiang University. Med. Sci. 49(1), 0–0 (2020). 10.3785/j.issn.1008-9292.2020.02.02 PMC8800711 32391658

[93] Deng L, Li C, Zeng Q Arbidol combined with LPV/r versus LPV/r alone against Corona Virus Disease 2019: a retrospective cohort study. J. Infect. (2020). (Epub ahead of print). 10.1016/j.jinf.2020.03.002 PMC7156152 32171872

[94] Clinicaltrials.Gov. Clinical Study of Arbidol Hydrochloride Tablets in the Treatment of Pneumonia Caused by Novel Coronavirus. (2020). https://clinicaltrials.gov/ct2/show/NCT04260594

[95] Moskal M, Beker W, Roszak R Suggestions for second-pass anti-COVID-19 drugs based on the artificial intelligence measures of molecular similarity, shape and pharmacophore distribution. (2020). 10.26434/chemrxiv.12084690.v2

[96] Kearney J Chloroquine as a potential treatment and prevention measure for the 2019 novel coronavirus: a review. 2020030275 (2020). .

[97] Gao J, Tian Z, Yang X Breakthrough: chloroquine phosphate has shown apparent efficacy in treatment of COVID-19 associated pneumonia in clinical studies. Bio. Sci. Trends. 14(1), 72–73; advpub (2020). . 10.5582/bst.2020.01047 32074550

[98] Sahraei Z, Shabani M, Shokouhi S, Saffaei A Aminoquinolines Against Coronavirus Disease 2019 (COVID-19): Chloroquine or Hydroxychloroquine. Int J Antimicrob Agents. 105945 (2020). 32194152 10.1016/j.ijantimicag.2020.105945 PMC7156117

[99] Philippe Gautreta J-CL, Philippe Parolaa, Van Thuan Hoang Hydroxychloroquine and azithromycin as a treatment of COVID-19: results of an open-label non-randomized clinical trial. International Journal of Antimicrobial Agents – In Press 17 March 2020. Int. J. Antimicrob. Agents 105949 (2020). 32205204 10.1016/j.ijantimicag.2020.105949 PMC7102549

[100] Liu J, Cao R, Xu M Hydroxychloroquine, a less toxic derivative of chloroquine, is effective in inhibiting SARS-CoV-2 infection *in vitro*. Cell. Dis. 6(1), 1–4 (2020). 10.1038/s41421-020-0156-0 PMC7078228 32194981

[101] Yao X, Ye F, Zhang M *In Vitro* Antiviral activity and projection of optimized dosing design of hydroxychloroquine for the treatment of severe acute respiratory syndrome coronavirus 2 (SARS-CoV-2). Clin. Infect. Dis. pii: ciaa237 (2020). (Epub ahead of print). 10.1093/cid/ciaa237 PMC7108130 32150618

[102] Srivatsan Padmanabhan M Potential dual therapeutic approach against SARS-CoV-2/COVID-19 with nitazoxanide and hydroxychloroquine.

[103] Chctr.Org. Clinical Trials on Chloroquine for SARS-COV-2 infection. (2020). http://www.chictr.org.cn/searchprojen.aspx

[104] Clinicaltrials.Gov. Clinical trials on chloroquine (2020). https://clinicaltrials.gov/ct2/results?cond=Covid+19&term=chloroquine&cntry=&state=&city=&dist=

[105] Clinicaltrials.Gov. Various Combination of Protease Inhibitors, Oseltamivir, Favipiravir, and Chloroquin for Treatment of COVID19 : A Randomized Control Trial (THDMS-COVID19). (2020). https://clinicaltrials.gov/ct2/show/NCT04303299

[106] Gordon DE, Jang GM, Bouhaddou M A SARS-CoV-2-human protein-protein interaction map reveals drug targets and potential drug-repurposing. BioRxiv 002386 (2020). 10.1038/s41586-020-2286-9 PMC7431030 32353859

[107] Devaux CA, Rolain JM, Colson P, Raoult D New insights on the antiviral effects of chloroquine against coronavirus: what to expect for COVID-19? Int. J. Antimicrob. Agents (2020). 10.1016/j.ijantimicag.2020.105938 PMC7118659 32171740

[108] Sarzi-Puttini P, Giorgi V, Sirotti S COVID-19, cytokines and immunosuppression: what can we learn from severe acute respiratory syndrome? Clin. Exp. Rheumatol. 38(2), 337–342 (2020). 32202240

[109] Conti P, Ronconi G, Caraffa A Induction of pro-inflammatory cytokines (IL-1 and IL-6) and lung inflammation by coronavirus-19 (COVI-19 or SARS-CoV-2): anti-inflammatory strategies. J. Biol. Reg. Homeos AG 34(2), (2020). (Epub ahead of print). 10.23812/CONTI-E 32171193

[110] Gabay C, Emery P, Van Vollenhoven R Tocilizumab monotherapy versus adalimumab monotherapy for treatment of rheumatoid arthritis (ADACTA): a randomised, double-blind, controlled phase 4 trial. Lancet 381(9877), 1541–1550 (2013). 23515142 10.1016/S0140-6736(13)60250-0

[111] Chisholm-Burns MA, Wells BG, Schwinghammer TL Pharmacotherapy Principles and Practice. McGraw-Hill, NY, USA (2016).

[112] Yokota S, Imagawa T, Mori M Efficacy and safety of tocilizumab in patients with systemic-onset juvenile idiopathic arthritis: a randomised, double-blind, placebo-controlled, withdrawal phase III trial. Lancet 371(9617), 998–1006 (2008). 18358927 10.1016/S0140-6736(08)60454-7

[113] Mehta P, Mcauley DF, Brown M, Sanchez E, Tattersall RS, Manson JJ COVID-19: consider cytokine storm syndromes and immunosuppression. Lancet 395(10229), P1033–1034 (2020). 10.1016/S0140-6736(20)30628-0 PMC7270045 32192578

[114] Xu X, Han M, Li T Effective treatment of severe COVID-19 patients with tocilizumab. Proc. Natl Acad. Sci. 202005615 . 10.1073/pnas.2005615117 PMC7245089 32350134

[115] Clinicaltrials.Gov. Tocilizumab in COVID-19 Pneumonia (TOCIVID-19) (TOCIVID-19). (2020). https://clinicaltrials.gov/ct2/show/NCT04317092?term=tocilizumab&cond=Covid+19&draw=2&rank=1

[116] Clinicaltrials.Gov. Tocilizumab for SARS-CoV2 Severe Pneumonitis (NCT04315480). (2020). https://clinicaltrials.gov/ct2/show/NCT04315480

[117] Chctr.Org. Favipiravir Combined With Tocilizumab in the Treatment of novel coronavirus pneumonia (COVID-19) - A Multicenter, Randomized, Controlled Trial (2020). www.chictr.org.cn/showprojen.aspx?proj=51126

[118] Gao J, Tian Z, Yang X Breakthrough: chloroquine phosphate has shown apparent efficacy in treatment of COVID-19 associated pneumonia in clinical studies. Biosci Trends 14(1), 72–73 (2020). 32074550 10.5582/bst.2020.01047

[119] Clinicaltrials.Gov. Evaluation of the efficacy and safety of sarilumab in hospitalized patients with COVID-19. (2020). https://clinicaltrials.gov/ct2/show/NCT04315298

[120] Assoun S, Brosseau S, Steinmetz C, Gounant V, Zalcman G Bevacizumab in advanced lung cancer: state of the art. Future Oncol. 13(28), 2515–2535 (2017). 28812378 10.2217/fon-2017-0302

[121] Clinicaltrials.Gov. The efficacy and safety of bevacizumab in severe or critical patients with COVID-19 pneumonia – a multi-centered randomized controlledclinical trial. (2020). https://clinicaltrials.gov/ct2/show/NCT04305106?term=Bevacizumab&cond=covid+19&draw=2&rank=1

[122] Wang Y, Jiang W, He Q Early, low-dose and short-term application of corticosteroid treatment in patients with severe COVID-19 pneumonia: single-center experience from Wuhan, China. MedRxiv. (2020) (Epub ahead of print).

[123] Zhou Y-H, Qin Y-Y, Lu Y-Q Effectiveness of glucocorticoid therapy in patients with severe novel coronavirus pneumonia: protocol of a randomized controlled trial. Chin. Med. J. (2020) (Epub ahead of print). 10.1097/CM9.0000000000000791 PMC7147272 32149773

[124] Clinicaltrials.Gov. Glucocorticoid therapy for novel coronavirus. (2020). https://clinicaltrials.gov/ct2/show/NCT04244591?term=Glucocorticoid&cond=COVID+19&draw=2&rank=1

[125] Doss M Treatment of COVID-19 with individualized immune boosting interventions. 2020030319, (2020).

[126] Caly L, Druce JD, Catton MG, Jans DA, Wagstaff KM The FDA-approved drug ivermectin inhibits the replication of SARS-CoV-2 *in vitro*. Antivir. Res. 178, 104787 (2020). 32251768 10.1016/j.antiviral.2020.104787 PMC7129059

[127] Patel A, Desai S Ivermectin in COVID-19 related critical illness. Available at SSRN 3570270 (2020). https://papers.ssrn.com/sol3/papers.cfm?abstract_id=3570270

[128] Patrì A, Fabbrocini G Hydroxychloroquine and ivermectin: a synergistic combination for COVID-19 chemoprophylaxis and/or treatment? .J. Am. Acad. Dermatol. PII: S0190-9622(20)30557-0 (2020) (Epub ahead of print). 10.1016/j.jaad.2020.04.017 PMC7146719 32283237

[129] Harrison C Coronavirus puts drug repurposing on the fast track. Nat. Biotechnol. 38(4), 379–381 (2020). . 32205870 10.1038/d41587-020-00003-1

[130] Chen L, Deng H, Cui H Inflammatory responses and inflammation-associated diseases in organs. Oncotarget 9(6), 7204–7218 (2018). 29467962 10.18632/oncotarget.23208 PMC5805548

[131] Zhou Y, Hou Y, Shen J, Huang Y, Martin W, Cheng F Network-based drug repurposing for novel coronavirus 2019-nCoV/SARS-CoV-2. Cell Discov. 6, 14 (2020). 32194980 10.1038/s41421-020-0153-3 PMC7073332

[132] Richardson P, Griffin I, Tucker C Baricitinib as potential treatment for 2019-nCoV acute respiratory disease. Lancet 395(10223), e30–e31 (2020). 32032529 10.1016/S0140-6736(20)30304-4 PMC7137985

[133] Tan D-X, Korkmaz A, Reiter RJ, Manchester LC Ebola virus disease: potential use of melatonin as a treatment. J. PIneal Res. 57(4), 381–384 (2014). 25262626 10.1111/jpi.12186

[134] Tan DX, Korkmaz A, Reiter RJ, Manchester LC Ebola virus disease: potential use of melatonin as a treatment. J. Pineal Res. 57(4), 381–384 (2014). 25262626 10.1111/jpi.12186

[135] Glowacka I, Bertram S, Herzog P Differential downregulation of ACE2 by the spike proteins of severe acute respiratory syndrome coronavirus and human coronavirus NL63. J. Virol. 84(2), 1198–1205 (2010). 19864379 10.1128/JVI.01248-09 PMC2798380

[136] Kruse RL Therapeutic strategies in an outbreak scenario to treat the novel coronavirus originating in Wuhan, China. F1000 Res. 9, 72 (2020). 10.12688/f1000research.22211.2 PMC7029759 32117569

[137] Wong SK, Li W, Moore MJ, Choe H, Farzan M A 193-amino acid fragment of the SARS coronavirus S protein efficiently binds angiotensin-converting enzyme 2. J. Biol. Chem. 279(5), 3197–3201 (2004). 14670965 10.1074/jbc.C300520200 PMC7982343

[138] Dong X, Fu J, Yin X Emodin: a review of its pharmacology, toxicity and pharmacokinetics. Phytother. Res. 30(8), 1207–1218 (2016). 27188216 10.1002/ptr.5631 PMC7168079

[139] Zhang D-H, Wu K-L, Zhang X, Deng S-Q, Peng B *In silico* screening of Chinese herbal medicines with the potential to directly inhibit 2019 novel coronavirus. J. Integr. Med. 18(2), 152–158 (2020). 32113846 10.1016/j.joim.2020.02.005 PMC7102521

[140] Liu X, Wang X-J Potential inhibitors for 2019-nCoV coronavirus M protease from clinically approved medicines. bioRxiv 47(2), 119–121 (2020). 10.1016/j.jgg.2020.02.001 PMC7128649 32173287

[141] Anand K, Ziebuhr J, Wadhwani P, Mesters JR, Hilgenfeld R Coronavirus main proteinase (3CLpro) structure: basis for design of anti-SARS drugs. Science 300(5626), 1763–1767 (2003). 12746549 10.1126/science.1085658

[142] Hsu M-F, Kuo C-J, Chang K-T Mechanism of the maturation process of SARS-CoV 3CL protease. J. Biol. Chem. 280(35), 31257–31266 (2005). 15788388 10.1074/jbc.M502577200 PMC8062786

[143] Yang Y, Islam MS, Wang J, Li Y, Chen X Traditional Chinese medicine in the treatment of patients infected with 2019-new coronavirus (SARS-CoV-2): a review and perspective. Int. J. Biol. Sci. 16(10), 1708–1717 (2020). 32226288 10.7150/ijbs.45538 PMC7098036

[144] Luo H, Tang Q-L, Shang Y-X Can Chinese medicine be used for prevention of corona virus disease 2019 (COVID-19)? A review of historical classics, research evidence and current prevention programs. Chin. J. Integr. Med. 26, 243–250 (2020). 32065348 10.1007/s11655-020-3192-6 PMC7088641

[145] Ren J, Zhang A-H, Wang X-J Traditional Chinese Medicine for COVID-19 Treatment. Pharmacol. Res. 155, 104743 (2020). . 32145402 10.1016/j.phrs.2020.104743 PMC7128263

[146] Chavez S, Long B, Koyfman A, Liang SY Coronavirus Disease (COVID-19): a primer for emergency physicians. Am. J. Emerg. Med. pii: S0735-6757(20)30178-9 (Epub ahead of print). 10.1016/j.ajem.2020.03.036 PMC7102516 32265065

[147] Jin Y-H, Cai L, Cheng Z-S A rapid advice guideline for the diagnosis and treatment of 2019 novel coronavirus (2019-nCoV) infected pneumonia (standard version). Military Medical Research. 7(1), 4 (2020). 32029004 10.1186/s40779-020-0233-6 PMC7003341

[148] Liu C, Zhou Q, Li Y Research and development on therapeutic agents and vaccines for COVID-19 and related human coronavirus diseases. ACS Central Sci. 6(3), 315–331 (2020). 10.1021/acscentsci.0c00272 PMC10467574 32226821

